# Differences by Race in Outcomes of an In-Person Training Intervention on Use of an Inpatient Portal: A Secondary Analysis of a Randomized Clinical Trial

**DOI:** 10.1001/jamanetworkopen.2024.5091

**Published:** 2024-04-04

**Authors:** Daniel M. Walker, Jennifer L. Hefner, Sarah R. MacEwan, Gennaro Di Tosto, Lindsey N. Sova, Alice A. Gaughan, Timothy R. Huerta, Ann Scheck McAlearney

**Affiliations:** 1 Department of Family and Community Medicine, College of Medicine, The Ohio State University, Columbus; 2 CATALYST, The Center for the Advancement of Team Science, Analytics, and Systems Thinking in Health Services and Implementation Science Research, College of Medicine, The Ohio State University, Columbus; 3 Division of Health Services Management and Policy, College of Public Health, The Ohio State University, Columbus; 4 Division of General Internal Medicine, College of Medicine, The Ohio State University, Columbus; 5 Department of Biomedical Informatics, College of Medicine, The Ohio State University, Columbus

## Abstract

**Importance:**

Differences in patient use of health information technologies by race can adversely impact equitable access to health care services. While this digital divide is well documented, there is limited evidence of how health care systems have used interventions to narrow the gap.

**Objective:**

To compare differences in the effectiveness of patient training and portal functionality interventions implemented to increase portal use among racial groups.

**Design, Setting, and Participants:**

This secondary analysis used data from a randomized clinical trial conducted from December 15, 2016, to August 31, 2019. Data were from a single health care system and included 6 noncancer hospitals. Participants were patients who were at least 18 years of age, identified English as their preferred language, were not involuntarily confined or detained, and agreed to be provided a tablet to access the inpatient portal during their stay. Data were analyzed from September 1, 2022, to October 31, 2023.

**Interventions:**

A 2 × 2 factorial design was used to compare the inpatient portal training intervention (touch, in-person [high] vs built-in video tutorial [low]) and the portal functionality intervention (technology, full functionality [full] vs a limited subset of functions [lite]).

**Main Outcomes and Measures:**

Primary outcomes were inpatient portal use, measured by frequency and comprehensiveness of use, and use of specific portal functions. A logistic regression model was used to test the association of the estimators with the comprehensiveness use measure. Outcomes are reported as incidence rate ratios (IRRs) for the frequency outcomes or odds ratios (ORs) for the comprehensiveness outcomes with corresponding 95% CIs.

**Results:**

Of 2892 participants, 550 (19.0%) were Black individuals, 2221 (76.8%) were White individuals, and 121 (4.2%) were categorized as other race (including African, American Indian or Alaska Native, Asian or Asian American, multiple races or ethnicities, and unknown race or ethnicity). Black participants had a significantly lower frequency (IRR, 0.80 [95% CI, 0.72-0.89]) of inpatient portal use compared with White participants. Interaction effects were not observed between technology, touch, and race. Among participants who received the full technology intervention, Black participants had lower odds of being comprehensive users (OR, 0.76 [95% CI, 0.62-0.91), but interaction effects were not observed between touch and race.

**Conclusions and Relevance:**

In this study, providing in-person training or robust portal functionality did not narrow the divide between Black participants and White participants with respect to their inpatient portal use. Health systems looking to narrow the digital divide may need to consider intentional interventions that address underlying issues contributing to this inequity.

**Trial Registration:**

ClinicalTrials.gov Identifier: NCT02943109

## Key Points

**Question:**

Does the effectiveness of patient training and portal functionality interventions implemented to increase patient portal use differ by racial groups?

**Findings:**

In a secondary analysis of a randomized clinical trial of 2892 participants, Black participants had lower frequency of portal use compared with White participants, but the in-person training (compared with a training video) and the full set of portal functions (compared with a limited set of functions) interventions were not different in Black individuals and White individuals at increasing inpatient portal use.

**Meaning:**

These findings suggest that despite evidence that in-person training and robust portal functionality increased use across participants of all races, Black individuals still used the portal less than White individuals.

## Teaser

This secondary analysis of a randomized clinical trial assesses differences of the effectiveness of patient training and portal functionality interventions implemented to impact portal use by race.

## Introduction

Inequities in the use of the internet and internet-enabled devices are a growing concern.^1,2^ The digital divide describes the gap between individuals who have access to digital technologies and those who do not because of social, political, and economic factors.^3^ Racial differences in the use of the internet and devices is of particular concern as health care is increasingly reliant on internet-based tools to engage individuals in both their health and health care.^4^ One such area of concern is the use of patient portals,^5,6,7,8,9,10,11^ which have become a primary conduit for health care organizations to provide patients with access to their electronic health record (EHR), and the ability to communicate with their care team. These concerns are well founded as inequities between Black individuals and White individuals in patient portal use have been documented in the literature.^12,13^

Patient portal use is influenced by structural-, organizational-, and individual-level factors.^14^ Structural and systemic issues include limited access to the internet and internet-enabled devices, inadequate internet infrastructure, and the prohibitive costs of internet subscription services or devices.^7,15,16,17^ Organizational factors include the technological resources to offer a patient portal and the incentives for staff members to prioritize patient portal use.^18,19^ Beyond technical issues, additional individual-level factors are also known to impact racial disparities in portal use, including health literacy, digital literacy, and perceptions of self-efficacy,^12,20,21,22,23,24,25^ which may contribute to lack of awareness, usability challenges, and low perceived usefulness of portals,^26,27,28^ thereby impacting portal use.

Investigators have explored interventions to increase patient engagement with portals, including having portals available in the hospital setting. One approach to engaging patients during hospitalization has been to offer access to a portal tailored to the inpatient experience (ie, inpatient portal) on hospital-provided devices (eg, tablet computers) using the hospital’s internet network, thereby supporting equitable use of portals during hospitalization by eliminating the structural barriers of internet access and device ownership. Furthermore, to address barriers associated with health literacy, digital literacy, and self-efficacy, patient portal training interventions have been suggested as a means to improve patients’ comfort and skills surrounding portal use, as well as encourage use of portal functions.^29,30,31,32,33^ The inpatient environment is a particularly opportune place to deliver such training because individuals are accessible by interventionists and may be open to learning how to use tools that can help them engage with their health care, especially in the face of their current health crisis.

Recent evidence from a randomized clinical trial demonstrated that in-person portal training and/or access to full functionality on an inpatient portal can increase use of an inpatient portal and patient satisfaction and involvement in their care,^34^ but whether inpatient training or access to more portal functionality impact the racial digital divide in portal use remains uncertain, particularly between Black individuals and White individuals. This study aims to determine if a training or a technology intervention were differentially associated with use of an inpatient portal across racial subgroups. This evidence will provide important information about interventions that may help address race-based differences in patient portal use.

## Methods

### Study Design

Our study leverages data collected as part of a randomized controlled trial (RCT)—the High-Tech and High-Touch (HT2) study—conducted across 6 hospitals that were part of a single academic health care system in the Midwest. The rationale, design,^35^ and primary study findings from the HT2 study have been published.^34^ The study used a 2-by-2 factorial design to concurrently test the individual and joint effects of 2 interventions: (1) touch: in-person (ie, high-touch) vs a video tutorial (ie, low-touch) training on how to use the inpatient portal; and (2) technology: a fully functional version of an inpatient portal (ie, full technology) vs a version with limited functionality (ie, lite technology). The full technology version of the inpatient portal included 10 functions (ie, Dining on Demand; Bedside Tutorial; To Learn; Happening Soon; Taking Care of Me; Messages; My Health; Notes; I Would Like; and MyChart [link to sign up for outpatient portal]). The lite technology version included only 3 functions (ie, dining on demand; bedside tutorial; to learn) (see eTable 1 in Supplement 1 for additional information about functions). A complete description of the intervention, including the approach to recruitment and data collection, is available in the study protocol (Supplement 2).

This secondary analysis of a randomized clinical trial presents results that were not prespecified in the original study protocol. The study was approved by the institutional review board of The Ohio State University. All patients provided written informed consent, including a release of access to their medical record. Our results follow the Consolidated Standards of Reporting Trials (CONSORT) reporting guideline. The original study protocol was updated in November 2023 to correct errors concerning protocol deviations that resulted in reallocation of participants who did not receive their originally allocated intervention.^36^ The revised protocol as well as the original protocol are included in Supplement 2.

### Study Population

The Ohio State University Wexner Medical Center offers standardized tablet computers to all patients admitted to any of its 6 noncancer hospitals who are at least 18 years, with a preferred language of English, and who are not involuntarily confined or detained. Patients can choose to decline the tablet. The tablet is equipped with the inpatient portal, MyChart Bedside (Epic Systems). The trial was conducted between December 2016 and August 2019.

**Figure 1.**
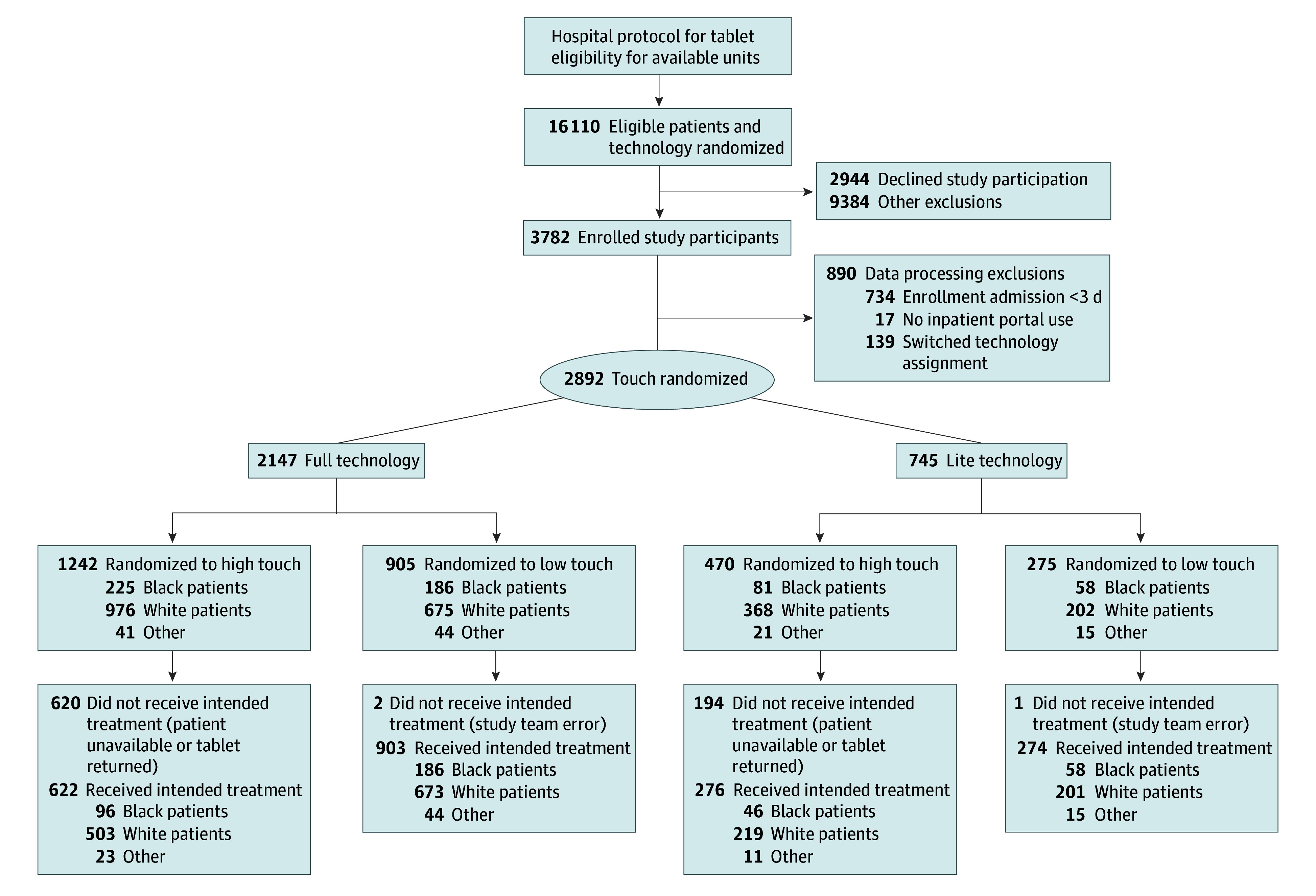
Enrollment and Randomization of Patients Patients were randomized to 1 of 4 groups: (1) full technology and high level of training (full technology, high-touch); (2) full technology and low level of training (full technology, low-touch); (3) less technology and high level of training (lite technology, high-touch); or (4) less technology and low level of training (lite technology, low-touch).

Patients who were hospitalized were eligible for the study if they agreed to receive a tablet and consented to study participation. Throughout the study, 2944 of the eligible study population declined to participate, and 9384 were excluded for reasons including patients receiving a tablet but being discharged before completion of study consent or HIPAA authorization or returning tablets before study introduction by research staff. Of the 3782 participants enrolled in the study, an additional 890 were excluded during data processing (ie, enrollment admission less than 3 days) (734 patients), no inpatient portal use (17 patients), and switched technology assignment following randomization (139 patients) resulting in 2892 participants randomized into the 4 study groups (Figure 1).

### Exposures

Three estimators of interest were assessed for this analysis: technology status, touch status, and racial subgroup. Technology and touch status were both used as dichotomous variables based on the study intervention group that was randomly assigned to each participant. The racial subgroup data was determined from patient self-report and extracted from the electronic health record (EHR) and included 3 levels: Black individuals, White individuals, and other individuals. The other group consisted of several racial groups that had a low frequency in the study population and included African, American Indian or Alaska Native, Asian or Asian American, multiple races or ethnicities, and unknown race or ethnicity.

### Outcomes

The primary outcomes of interest were frequency and comprehensiveness of inpatient portal use, which were derived from audit log files associated with the patients’ inpatient portal use during their hospital stay in which they received the touch intervention.^37^ The frequency of inpatient portal use was defined as the number of unique sessions during the patient’s hospital stay in which they were enrolled in the study. A binary measure of comprehensiveness of inpatient portal use was determined by the breadth of functions used by a participant and differed by technology treatment status: lite technology participants were defined as a comprehensive user if they used all 3 lite technology functions; full technology participants were defined as a comprehensive user if they used 8 or more functions.

### Statistical Analysis

The analytic approach for this secondary analysis of the HT2 study aimed to test the differential effects of the technology and touch interventions, both individually and in combination, on the outcomes across racial subgroups. All analyses were conducted using both an intention-to-treat (ITT) framework where participants are analyzed in their randomly assigned study groups, as well as a per-protocol specification where only participants who received their allocated intervention were included in the analysis. The associations of the study interventions with inpatient portal session frequency were tested using a negative binomial model with noninteracted and interacted study groups and the racial subgroups as estimators. A logistic regression model was used to test the association of the estimators with the comprehensiveness use measure. We then estimated and plotted the number of sessions and the probability of being a comprehensive user for each racial group in each study group to improve interpretability of interactions. We further analyzed the inpatient portal use outcomes in the full technology subsample alone and conducted an exploratory test of the association of the touch intervention and racial subgroup with each function of inpatient portal using a fractional logistic regression model. For all analyses, low-touch, lite technology, and White patients are used as reference categories.

All available data were used for the analyses. Outcomes are reported as incidence rate ratios (IRRs) for the frequency outcomes or odds ratios (ORs) for the comprehensiveness outcomes with corresponding 95% CIs. To account for heterogeneity in patient characteristics across the 6 hospitals, all analyses include cluster-robust standard errors based on facility at study enrollment. Further, to account for heterogeneity in duration of patient access to the inpatient portal, we included the length of tablet provisioning, measured in days, as an offset in all models. Per-protocol analyses included inverse probability weights to account for selection bias associated with receipt of the intervention compared with those who did not receive the intervention.^38^ All analyses were conducted using Stata MP version 17.0 (StataCorp) and occurred from September 1, 2022, to October 31, 2023.

## Results

Of the 2892 participants (median [IQR] age, 47 [35-59] years; 1641 [56.7%] female, 1251 [43.4%] male) randomized into the study sample, 550 (19.0%) were Black patients, 2221 (76.8%) were White patients, and 121 (4.2%) were categorized as other race (Table 1 and eTable 2 in Supplement 1). Most respondents across each study group and racial group were female, with the exception of the other group in the lite-technology and low-touch group where a greater percentage was male. The highest median (IQR) age was observed in the White group in 3 of the study groups: high-technology and low-touch (49.0 [34.0-60.0] years); lite-technology and high-touch (49.0 [36.0-61.0] years); lite-technology and low-touch (49.0 [37.0-60.0] years), and the other group in the lite-technology and high-touch group had the lowest median ages (37.0 [29.0-42.0] years).

**Table 1.** Demographic and Clinical Characteristics for Patients at Enrollment Admission (Intention-to-Treat), by Study Group Assignment and Race

Characteristic	Study group and race, No. (%)											
	Full technology						Lite technology					
	High touch			Low touch			High touch			Low touch		
	Black (n = 225)	White (n = 976)	Other^a^ (n = 41)	Black (n = 186)	White (n = 675)	Other^a^ (n = 44)	Black (n = 81)	White (n = 368)	Other^a^ (n = 21)	Black (n = 58)	White (n = 202)	Other^a^ (n = 15)
Gender												
Female	134 (59.6)	550 (56.4)	25 (61.0)	109 (58.6)	382 (56.6)	30 (68.2)	44 (54.3)	199 (54.1)	13 (61.9)	35 (60.3)	113 (55.9)	7 (46.7)
Male	91 (40.4)	426 (43.6)	16 (39.0)	77 (41.4)	293 (43.4)	14 (31.8)	37 (45.7)	169 (45.9)	8 (38.1)	23 (39.7)	89 (44.1)	8 (53.3)
Age at enrollment, median (IQR), y	47.0 (35.0-57.0)	47.0 (34.0-58.0)	40.0 (29.0-54.0)	45.0 (35.0-57.0)	49.0 (34.0-60.0)	45.0 (34.5-54.0)	48.0 (35.0-57.0)	49.0 (36.0-61.0)	37.0 (29.0-42.0)	45.0 (32.0-58.0)	49.0 (37.0-60.0)	48.0 (36.0-53.0)
Charlson Comorbidity Index, median (IQR)	2.0 (1.0-3.0)	1.0 (0-3.0)	1.0 (0-2.0)	2.0 (1.0-3.0)	2.0 (0-3.0)	2.0 (1.0-3.0)	2.0 (0-4.0)	1.0 (0-3.0)	1.0 (0-2.0)	2.0 (0-3.0)	2.0 (0-3.0)	1.0 (0-2.0)
Length of stay, median (IQR), d	6.0 (4.0-10.0)	6.0 (4.0-11.0)	6.0 (4.0-14.0)	6.0 (4.0-9.0)	6.0 (4.0-12.0)	8.5 (5.0-15.5)	7.0 (4.0-11.0)	6.0 (4.0-12.0)	5.0 (3.0-6.0)	5.0 (4.0-10.0)	7.0 (4.0-12.0)	6.0 (3.0-9.0)
Length of provisioning, median (IQR), d^b^	6.0 (4.0-9.0)	6.0 (4.0-10.0)	6.0 (4.0-15.0)	5.0 (4.0-8.0)	6.0 (4.0-10.0)	8.0 (5.0-14.5)	7.0 (4.0-9.0)	6.0 (4.0-9.5)	5.0 (4.0-6.0)	5.5 (4.0-9.0)	6.0 (4.0-10.0)	5.0 (3.0-8.0)
MyChart account	127 (56.4)	686 (70.3)	30 (73.2)	110 (59.1)	439 (65.0)	34 (77.3)	36 (44.4)	223 (60.6)	11 (52.4)	21 (36.2)	111 (55.0)	10 (66.7)
MyChart frequency of use within 3 mo prior to study enrollment, median (IQR)^c^	0 (0-6.0)	0 (0-10.0)	1.0 (0-14.0)	0 (0-5.0)	0 (0-13.0)	0 (0-10.5)	0	0 (0-11.0)	0 (0-7.0)	0	0 (0-10.0)	0 (0-12.0)

^a^ Other includes African, American Indian or Alaska Native, Asian or Asian American, multiple races or ethnicities, and unknown race or ethnicity.

^b^ Length of provisioning is defined as the number of days the patient had possession of the tablet.

^c^ MyChart frequency of use is measured by a count of number of log-in sessions (Supplement 2).

### Combined Outcomes of Full Technology and High-Touch Interventions and Race on Inpatient Portal Use

Model results from the analysis of the combined effects of the technology and touch interventions on inpatient portal use, including the interaction with the race categories, are presented in Table 2. In the ITT analysis, participants who received the full technology intervention compared with those in the lite technology group had a significantly higher frequency (IRR, 1.42 [95% CI, 1.19-1.71]) of inpatient portal use and lower odds of being classified as a comprehensive inpatient portal user (ie, used all 3 lite technology functions or 8 or more full technology functions) (OR, 0.61 [95% CI, 0.43-0.88]). Participants in the high-touch intervention compared with those in the low-touch group had greater odds of being a comprehensive user (OR, 2.88 [95% CI, 2.14-3.87]). In the per-protocol analysis, results were similar for the full technology intervention for both frequency and comprehensiveness, and participants receiving the high-touch intervention were more frequent users (IRR, 1.13 [95% CI, 1.01-1.26]) and had amplified odds of being comprehensive users (OR, 13.02 [95% CI, 8.59-19.73]), and there was a significant interaction for comprehensiveness in the full-technology and high-touch group (OR, 1.97 [95% CI, 1.04-3.72]).

**Table 2.** Inpatient Portal Use Outcomes, by Study Group Assignment and Interaction With Race

Treatment and Level	Frequency				Comprehensiveness			
	ITT, mean (SD)	ITT, IRR (95% CI)	PP, mean (SD)	PP, IRR (95% CI)	ITT, No. (%)^a^	ITT, OR (95% CI)^a^	PP, No. (%)^a^	PP, OR (95% CI)^a^
Technology								
Lite	20.4 (21.0)	1 [Reference]	21.6 (22.0)	1 [Reference]	396 (53.1)	1 [Reference]	348 (63.3)	1 [Reference]
Full	29.3 (32.9)	1.42 (1.19-1.71)	32.1 (35.1)	1.43 (1.20-1.72)	950 (44.2)	0.61 (0.43-0.88)	1525 (52.8)	0.62 (0.42-0.90)
Touch								
Low	27.2 (33.8)	1 [Reference]	27.1 (33.8)	1 [Reference]	357 (30.2)	1 [Reference]	355 (30.2)	1 [Reference]
High	26.8 (28.1)	1.05 (0.92-1.20)	32.2 (30.4)	1.13 (1.01-1.26)	989 (57.8)	2.88 (2.14-3.87)	798 (88.9)	13.02 (8.59-19.73)
Technology × touch								
Full and high	NA	1.06 (0.85-1.31)	NA	1.06 (0.87-1.30	NA	1.34 (0.98-1.84)	NA	1.97 (1.04-3.72)
Race								
Black	19.4 (18.7)	0.80 (0.72-0.89)	21.1 (20.1)	0.81 (0.73-0.89)	207 (37.6)	0.60 (0.18-1.99)	175 (45.3)	0.61 (0.18-2.04)
White	28.7 (31.8)	1 [Reference]	31.0 (33.6)	1 [Reference]	1080 (48.6)	1 [Reference]	926 (58.0)	1 [Reference]
Other^b^	30.6 (42.2)	0.98 (0.65-1.48)	33.8 (46.6)	0.98 (0.65-1.48)	59 (48.7)	1.86 (0.72-4.84)	52 (55.9)	1.91 (0.75-4.84)
Technology × race								
Technology × Black	NA	1.12 (0.88-1.43)	NA	1.12 (0.88-1.42)	NA	1.26 (0.32-4.92)	NA	1.28 (0.32-5.18)
Technology × White	NA	1 [Reference]	NA	1 [Reference]	NA	1 [Reference]	NA	1 [Reference]
Technology × other	NA	0.99 (0.63-1.57)	NA	0.98 (0.63-1.53)	NA	0.39 (0.10-1.55)	NA	0.39 (0.10-1.53)
Touch × race								
Touch × Black	NA	1.10 (0.87-1.38)	NA	1.12 (0.88-1.43)	NA	1.44 (0.40-5.25)	NA	1.73 (0.62-4.83)
Touch × White	NA	1 [Reference]	NA	1 [Reference]	NA	1 [Reference]	NA	1 [Reference]
Touch × other	NA	1.06 (0.60-1.89)	NA	1.07 (0.56-2.05)	NA	0.61 (0.21-1.77)	NA	1.31 (0.47-3.66)
Technology × touch × race								
Full, high, and Black	NA	0.74 (0.48-1.14)	NA	0.73 (0.43-1.24)	NA	0.55 (0.13-2.38)	NA	0.31 (0.07-1.43)
Full, high, and White	NA	1 [Reference]	NA	1 [Reference]	NA	1 [Reference]	NA	1 [Reference]
Full, high, and other	NA	0.81 (0.44-1.49)	NA	0.80 (0.43-1.52)	NA	2.60 (0.94-7.16)	NA	1.00 (1.00-1.00)^c^

Abbreviations: IRR, incidence rate ratio; ITT, intention-to-treat; OR, odds ratio; PP, per protocol.

^a^ A comprehensive portal user was one who used all 3 lite-technology functions (for lite-technology participants) or used 8 or more of the 10 full technology functions (for full technology participants).

^b^ Other includes African, American Indian or Alaska Native, Asian or Asian American, multiple races or ethnicities, and unknown race and ethnicity.

^c^ All other participants in this group were comprehensive users, resulting in the inability to estimate an IRR for this group.

For the main outcomes associated with race, participants who were Black had a significantly lower frequency (IRR, 0.80 [95% CI, 0.72-0.89]) of inpatient portal use compared with White participants, and this result was similar in the per-protocol analysis. Focusing on the interaction between technology, touch, and race, no significance was observed for either frequency or comprehensiveness in either the ITT or per-protocol analyses.

### Association of Touch Intervention and Race Among Participants Receiving Full Technology Intervention

**Figure 2.**
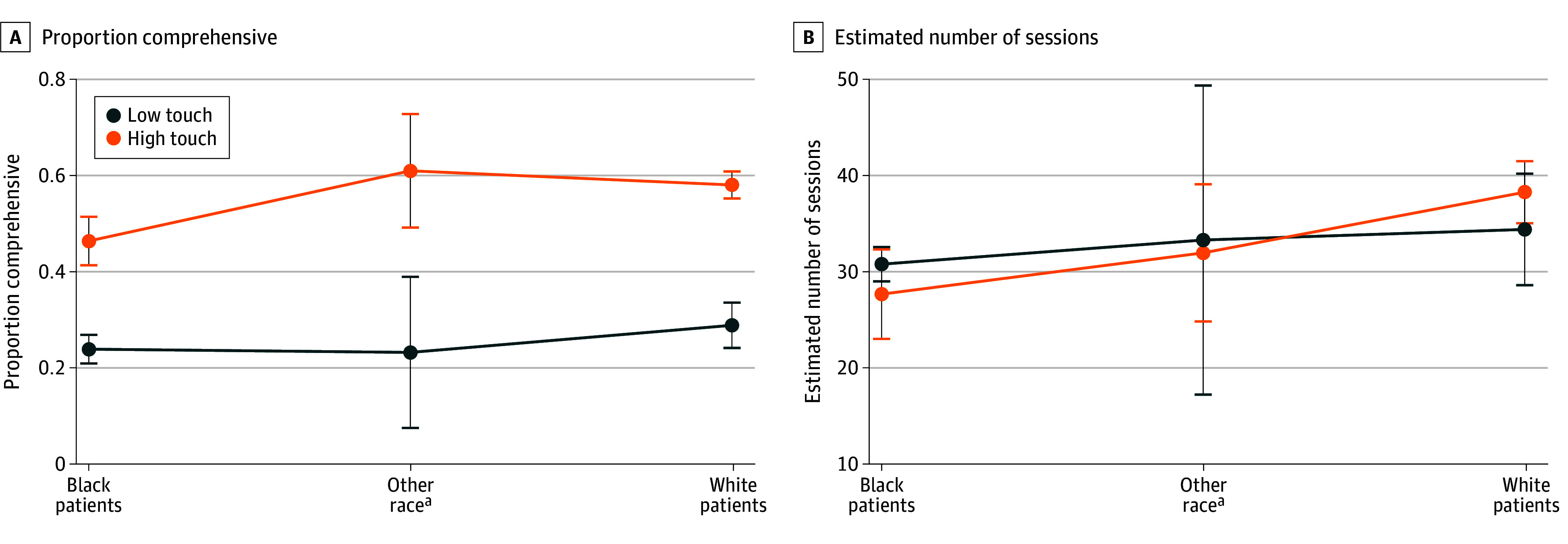
Frequency and Comprehensiveness of Inpatient Portal Use in the Full Technology Subsample by Touch Status and Race Group. ^a^Other includes African, American Indian or Alaska Native, Asian or Asian American, multiple races or ethnicities, and unknown race and ethnicity.

Interaction plots of a subsample analysis of inpatient portal use limited to participants in the full technology study group are displayed in Figure 2 (ITT only). Full model results are available in eTable 3 in Supplement 1. Black participants had lower odds of being comprehensive users (ITT: OR, 0.76 [95% CI, 0.62-0.91]; per-protocol: OR, 0.78 [95% CI, 0.64-0.95]). The interaction between touch and race was not significant for the Black participants or other participants for either frequency or comprehensiveness outcomes in either the ITT or per-protocol models.

Within the full technology subsample, we further explored use of each inpatient portal function (Table 3 and eTable 4 in Supplement 1). In the ITT analysis, the effect of touch on the odds of a participant using the Taking Care of Me function was greater in Black individuals relative to White individuals (OR, 1.49 [95% CI, 1.13-1.95]). The odds of participants categorized as other in the high-touch group using the To Learn (OR, 2.52 [95% CI, 1.28-4.96]), Notes (OR, 3.08 [95% CI, 1.24-7.63]), and I Would Like (OR, 6.90 [95% CI, 1.41-33.85]) functions were greater relative to White participants in the low-touch group. In the per-protocol analysis, results were similar for Black participants. However, for participants categorized as other, significance was no longer observed for the I Would Like function, and a significant difference was observed for Dining on Demand (OR, 1.67 [95% CI, 1.07-2.60]).

**Table 3.** Subsample Analysis of Inpatient Portal Use Outcomes for Participants in the Full Technology Study Group (Intention-to-Treat), by Touch Status and Race

Outcome	Treatment							
	Touch		Race			Touch × race		
	Low	High	Black	White	Other^a^	Touch × Black	Touch × White	Touch × other
**Dining on Demand**								
Mean proportion (SD)	0.22 (0.20)	0.20 (0.17)	0.22 (0.19)	0.20 (0.18)	0.23 (0.17)	NA	NA	NA
OR	1 [Reference]	0.89 (0.77-1.04)	1.28 (1.14-1.43)	1 [Reference]	0.81 (0.70-0.93)	1.03 (0.84-1.26)	1 [Reference]	1.25 (0.74-2.11)
**Tutorial**								
Mean proportion (SD)	0.19 (0.22)	0.16 (0.18)	0.22 (0.23)	0.16 (0.19)	0.17 (0.18)	NA	NA	NA
OR	1 [Reference]	0.80 (0.73-0.86)	1.61 (1.15-1.63)	1 [Reference]	0.67 (0.44-1.03)	1.11 (0.73-1.68)	1 [Reference]	1.40 (0.68-2.89)
**To Learn**								
Mean proportion (SD)	0.01 (0.05)	0.02 (0.04)	0.02 (0.05)	0.02 (0.04)	0.01 (0.03)	NA	NA	NA
OR	1 [Reference]	1.37 (1.24-1.51)	1.34 (0.80-2.25)	Ref	0.36 (0.16-0.76)	1.08 (0.40-2.89)	Ref	2.52 (1.28-4.96)
**My Health**								
Mean proportion (SD)	0.10 (0.17)	0.11 (0.15)	0.07 (0.13)	0.11 (0.17)	0.12 (0.18)	NA	NA	NA
OR	1 [Reference]	1.20 (1.09-1.31)	0.80 (0.56-1.14)	1 [Reference]	0.62 (0.38-1.01)	0.77 (0.58-1.01)	1 [Reference]	1.60 (0.72-3.58)
**Happening Soon**								
Mean proportion (SD)	0.37 (0.25)	0.39 (0.23)	0.37 (0.24)	0.39 (0.24)	0.36 (0.24)	NA	NA	NA
OR	1 [Reference]	1.15 (1.02-1.30)	1.12 (0.85-1.48)	1 [Reference]	0.77 (0.73-0.81)	0.81 (0.63-1.05)	1 [Reference]	0.82 (0.64-1.05)
**Notes**								
Mean proportion (SD)	0	0	0	0	0	NA	NA	NA
OR	1 [Reference]	1.15 (0.57-2.32)	0.63 (0.16-2.53)	1 [Reference]	0.30 (0.14-0.63)	1.14 (0.33-3.88)	1 [Reference]	3.08 (1.24-7.63)
**Messages**								
Mean proportion (SD)	0.03 (0.05)	0.03 (0.04)	0.03 (0.04)	0.03 (0.04)	0.03 (0.04)	NA	NA	NA
OR	1 [Reference]	1.03 (0.88-1.19)	0.95 (0.48-1.89)	1 [Reference]	0.48 (0.18-1.30)	1.24 (0.78-1.97)	1 [Reference]	2.14 (0.66-6.99)
**I Would Like**								
Mean proportion (SD)	0 (0.01)	0 (0.01)	0 (0.01)	0 (0.01)	0 (0.01)	NA	NA	NA
OR	1 [Reference]	1.96 (1.53-2.51)	1.03 (0.31-3.43)	1 [Reference]	0.17 (0.05-0.51)	0.97 (0.26-3.61)	1 [Reference]	6.90 (1.41-33.85)
**My Chart**								
Mean proportion (SD)	0.03 (0.04)	0.03 (0.04)	0.03 (0.04)	0.03 (0.04)	0.03 (0.05)	NA	NA	NA
OR	1 [Reference]	1.23 (1.09-1.38)	1.05 (0.58-1.91)	1 [Reference]	0.59 (0.20-1.73)	0.90 (0.52-1.56)	1 [Reference]	1.93 (0.64-5.77)
**Taking Care of Me**								
Mean proportion (SD)	0.04 (0.05)	0.05 (0.04)	0.04 (0.04)	0.05 (0.05)	0.04 (0.04)	NA	NA	NA
OR	1 [Reference]	1.09 (0.99-1.21)	0.76 (0.51-1.13)	1 [Reference]	0.60 (0.28-1.31)	1.49 (1.13-1.95)	1 [Reference]	1.36 (0.59-3.18)

Abbreviations: NA, not applicable; OR, odds ratio.

^a^ Other includes African, American Indian or Alaska Native, Asian or Asian American, multiple races or ethnicities, and unknown race or ethnicity.

## Discussion

The aim of this secondary analysis was to examine if the technology and touch interventions help overcome barriers related to access, usability, and perceived utility of patient portals in the inpatient setting. However, this secondary analysis focused on the interventions’ differential associations with race and found that the interventions did not close the gap in use of this health information technology tool. Moreover, our analysis found that Black individuals used the inpatient portal less frequently than White individuals. This finding suggests that the outcomes of the technology and touch interventions promoting greater portal use overall were not strong enough to foster greater use among Black participants.

Taken in combination, our findings suggest that the technology and touch interventions may have provided technology access as well as skills and digital literacy training,^39^ but did not overcome other barriers that disproportionately impact Black individuals. These additional barriers to inpatient portal use may include medical mistrust, privacy concerns, and/or unwillingness to share information electronically.^40,41^ Furthermore, the use case for patients to engage with their health care through a patient portal may be unclear, which may ultimately mediate the efficacy of the interventions.^42^

Patients’ perspectives about the usefulness of patient portals have likely changed significantly since the data collection period of this study. Specifically, the COVID-19 pandemic accelerated the expansion of telehealth as a means to receive care remotely^43^ and influenced policy changes to increase access to telehealth (eg, Medicare’s continuing coverage of telehealth for mental or behavioral health care).^44^ Patient portals now often serve as a platform for telehealth, such as in conducting synchronous video visits, and thus increasing a patient’s comfort using patient portals may also increase their willingness to use telehealth.^45^ Furthermore, recent policies in the United States are seeking to increase patients’ access to the internet and their personal health information. For example, the Infrastructure Investment and Jobs Act (ie, Bipartisan Infrastructure Bill), signed into law in 2021, included $65 billion to improve broadband internet access.^46^ Additionally, the 21st Century Cures Act, signed into law in 2016, has served to increase patients’ access to personal health information, including increasing timely and free access to personal information as of October 2022 due to expansion in the definition of electronic protected health information. These changes impact the information that can be shared via patient portals.^47^ For these reasons, as patient use of health information technologies likely continues to grow, disparities in the use of these tools must be addressed to ensure certain patient populations are not left behind.

### Limitations

This study has limitations. The racial subgroup of Black individuals identified from the EHR may be flawed and may not accurately reflect racial or ethnic identification, such as Black Hispanic or other African descent. Similarly, we needed to combine several racial subgroups into an other group due to low frequencies in our data, limiting the interpretability of findings from this group. In addition, racial groups serve as a proxy for other cultural or socioeconomic factors, and thus do not directly address questions about why differences in patient portal use between racial subgroups might persist. The well-documented racial differences in adoption of consumer-facing health information technologies have important implications for achieving health equity. Notably, inequities may stem from systemic racism, and thus disparities may reflect both the lived experiences of the individuals categorized as different races as well as the implicit biases of health care clinicians. Furthermore, the technology and touch interventions tested in the RCT were not designed to close the digital divide in technology acceptance and use among different race groups.

Our study was also not designed with health equity as a goal and did not aim to test the combined effects of the interventions by race. Thus, we did not have prespecified recruitment targets to identify racial differences and may, as a result, be underpowered to detect differences in study outcomes by race groups. Similarly, our interventions did not purposefully incorporate approaches that may help advance health equity. For instance, bias, both implicit and explicit, has been noted as a barrier in patient portal uptake among Black individuals,^48,49,50^ suggesting that it may be important to address this factor. Additionally, racial concordance of study staff delivering the touch intervention might improve patient trust and thereby differentially impact Black participants.^51^ Finally, training study staff in motivational interviewing^52^ could also increase the impact of the intervention on patients who may be hesitant to use the inpatient portal due to medical mistrust. Each of these approaches could be strengthened by codesign of a training intervention, whereby Black and other minoritized individuals are engaged in the design of the intervention prior to its implementation.^14,53,54^ Future health equity-focused technology training interventions may consider using these tactics.

## Conclusions

In this study, we found that the technology and touch interventions did not have different associations with inpatient portal use in participants of different racial subgroups. This suggests that to narrow the digital divide, focus is needed not only on portal training but also on developing culturally sensitive trainings that are targeted specifically to an individual’s needs and that present a compelling case for portal use. Implementing such trainings may be critical to increase the adoption of patient portals among Black individuals and can be an important step toward closing the digital divide.

## Notes

Supplement 1. **eTable 1.** Inpatient Portal Functions **eTable 2.** Demographic and Clinical Characteristics for Patients at Enrollment Admission Per Protocol, by Study Group Assignment and Race **eTable 3. **Subsample Analysis of Inpatient Portal Use Outcomes for Participants in the Full Technology Study Group, by Touch Status and Race **eTable 4.** Subsample Analysis of Inpatient Portal Use Outcomes for Participants in the Full Technology Study Group Per Protocol, by Touch Status and Race

Supplement 2. **eAppendix 1.** Trial Protocol **eAppendix 2.** Original Protocol

Supplement 3.
Data Sharing Statement
