# Supplementary material for: Differences by Race in Outcomes of an In-Person Training Intervention on Use of an Inpatient Portal: A Secondary Analysis of a Randomized Clinical Trial
Source: JAMA Netw Open. 2024 Apr 4;7(4):e245091. doi: 10.1001/jamanetworkopen.2024.5091 (PMC11192182; doi:10.1001/jamanetworkopen.2024.5091)
Supplement: Supplement 1. — eTable 1. Inpatient Portal Functions eTable 2. Demographic and Clinical Characteristics for Patients at Enrollment Admission Per Protocol, by Study Group Assignment and Race eTable 3. Subsample Analysis of Inpatient Portal Use Outcomes for Participants in the Full Technology Study Group, by Touch Status and Race eTable 4. Subsample Analysis of Inpatient Portal Use Outcomes for Participants in the Full Technology Study Group Per Protocol, by Touch Status and Race [file jamanetwopen-e245091-s001.pdf]

Supplemental Online Content

Walker DM, Hefner JL, MacEwan SR, et al. Differences by race in effectiveness of an in-person training intervention on use of an inpatient portal: a secondary analysis of a pragmatic randomized clinical trial. *JAMA Netw Open*. 2024;7(4):e245091.
doi:10.1001/jamanetworkopen.2024.5091

eTable 1. MyChart Bedside Inpatient Portal Functions

eTable 2. Demographic and Clinical Characteristics for Patients at Enrollment Admission Per Protocol, by Study Group Assignment and Race

eTable 3. Subsample Analysis of Inpatient Portal Use Outcomes for Participants in the Full-Technology Study Group, by Touch Status and Race

eTable 4. Subsample Analysis of Inpatient Portal Use Outcomes for Participants in the Full-Technology Study Group Per Protocol, by Touch Status and Race

This supplemental material has been provided by the authors to give readers additional information about their work.

eTable 1: MyChart Bedside inpatient portal functions.

**MyChart Bedside Function**	**Description**
Dining On Demand*	Order a meal from a predefined menu
Tutorial*	Access tutorials on the use of MyChart Bedside
To Learn*	Access training materials through a link to an external health information content provider
My Health	Review vitals and laboratory test results
Happening Soon	Review scheduled upcoming interactions with the care team
Notes	Record and review personal notes (audio and written)
Messages	Send and review secure messages
I Would Like	Request one of a number of ancillary services
MyChart	Create an outpatient MyChart account or change the password on an existing MyChart account
Taking Care of Me	Review active members of the care team

* Function available in Lite-Tech version of MyChart Bedside

© 2024 Walker DM et al. *JAMA Network Open.*

eTable 2. Demographic and clinical characteristics for patients at enrollment admission per-protocol, by study arm assignment and race.

	Study Group and Race[a]											
	Full-tech						Lite-tech					
	High-touch			Low-touch			High-touch			Low-touch		
**Characteristic**	**Black (n=96)**	**White (n=503)**	**Other[b] (n=23)**	**Black (n=186)**	**White (n=673)**	**Other[b] (n=44)**	**Black (n=46)**	**White (n=219)**	**Other[b] (n=11)**	**Black (n=58)**	**White (n=201)**	**Other[b] (n=15)**
**Gender**												
Female	52 (54.2%)	293 (58.3%)	13 (56.5%)	109 (58.6%)	382 (56.8%)	30 (68.2%)	24 (52.2%)	123 (56.2%)	4 (36.4%)	35 (60.3%)	112 (55.7%)	7 (46.7%)
Male	44 (45.8%)	210 (41.7%)	10 (43.5%)	77 (41.4%)	291 (43.2%)	14 (31.8%)	22 (47.8%)	96 (43.8%)	7 (63.6%)	23 (39.7%)	89 (44.3%)	8 (53.3%)
**Age at enrollment**												
Median (Q1, Q3)	49.0 (36.0, 56.0)	47.0 (36.0, 58.0)	39.0 (29.0, 48.0)	45.0 (35.0, 57.0)	49.0 (34.0, 60.0)	45.0 (34.5, 54.0)	42.5 (29.0, 59.0)	50.0 (38.0, 61.0)	39.0 (26.0, 42.0)	45.0 (32.0, 58.0)	49.0 (37.0, 60.0)	48.0 (36.0, 53.0)
**Charlson Comorbidity Index**												
Median (Q1, Q3)	2.0 (1.0, 3.0)	1.0 (0.0, 3.0)	1.0 (0.0, 2.0)	2.0 (1.0, 3.0)	2.0 (0.0, 3.0)	2.0 (1.0, 3.0)	2.0 (0.0, 3.0)	2.0 (0.0, 3.0)	0.0 (0.0, 1.0)	2.0 (0.0, 3.0)	2.0 (0.0, 3.0)	1.0 (0.0, 2.0)
**Length of stay (days)**												
Median (Q1, Q3)	6.5 (4.5, 11.0)	8.0 (4.0, 13.0)	7.0 (4.0, 16.0)	6.0 (4.0, 9.0)	6.0 (4.0, 12.0)	8.5 (5.0, 15.5)	8.0 (4.0, 14.0)	7.0 (5.0, 13.0)	6.0 (4.0, 7.0)	5.0 (4.0, 10.0)	7.0 (4.0, 12.0)	6.0 (3.0, 9.0)
**Length of provisioning[c] (days)**												
Median (Q1, Q3)	6.5 (4.5, 10.5)	7.0 (5.0, 11.0)	8.0 (4.0, 17.0)	5.0 (4.0, 8.0)	6.0 (4.0, 10.0)	8.0 (5.0, 14.5)	7.0 (5.0, 12.0)	6.0 (4.0, 12.0)	6.0 (4.0, 8.0)	5.5 (4.0, 9.0)	6.0 (4.0, 10.0)	5.0 (3.0, 8.0)
**MyChart account**												
MyChart account	58 (60.4%)	366 (72.8%)	17 (73.9%)	110 (59.1%)	439 (65.2%)	34 (77.3%)	21 (45.7%)	134 (61.2%)	5 (45.5%)	21 (36.2%)	110 (54.7%)	10 (66.7%)
**MyChart frequency of use[d] within 3 months prior to study enrollment**												
Median (Q1, Q3)	0.0 (0.0, 6.0)	0.0 (0.0, 12.0)	0.0 (0.0, 14.0)	0.0 (0.0, 5.0)	0.0 (0.0, 13.0)	0.0 (0.0, 10.5)	0.0 (0.0, 2.0)	0.0 (0.0, 13.0)	0.0 (0.0, 0.0)	0.0 (0.0, 0.0)	0.0 (0.0, 10.0)	0.0 (0.0, 12.0)

[a]Unless otherwise indicated, data are expressed as No. (%) of patients.

[b]Other includes African, American Indian or Alaska Native, Asian or Asian American, multiple races/ethnicities, and unknown race/ethnicity.

[c]Length of provisioning is defined as the number of days the patient had possession of the tablet.

[d]MyChart frequency of use is measured by a count of number of log-in sessions (see Statistical Analysis Plan for details).

© 2024 Walker DM et al. *JAMA Network Open.*

eTable 3: Sub-sample analysis of inpatient portal use outcomes for participants in the full-tech study arm, by touch status and race.

		Frequency				Comprehensiveness			
**Treatment**	**Level**	**ITT (mean, SD)**	**ITT (IRR)**	**PP (mean, SD)**	**PP (IRR)**	**ITT (n, %)**	**ITT (OR)**	**PP (n, %)**	**PP (OR)**
**Touch**	Low	29.3, 35.6	Ref.	27.1, 33.8	Ref.	253 (26.0%)	Ref.	252 (27.9%)	Ref.
	High	29.2, 30.8	**1.11 (1.01,1.23)**	32.2, 30.4	**1.20 (1.08,1.34)**	697 (88.8%)	**3.86 (3.10,4.81)**	553 (88.9%)	**25.67 (14.08,46.79)**
**Race**	Black	20.2, 19.3	0.90 (0.77,1.04)	21.1, 20.1	0.90 (0.77,1.05)	142 (34.5%)	**0.76 (0.62,0.91)**	118 (41.8%)	**0.78 (0.64,0.95)**
	White	31.1, 34.2	Ref.	31.0, 33.6	Ref.	769 (46.6%)	Ref.	653 (55.5%)	Ref.
	Other[b]	36.8, 48.4	0.97 (0.68,1.39)	33.8, 46.6	0.97 (0.68,1.37)	39 (45.9%)	0.73 (0.28,1.86)	34 (50.7%)	0.75 (0.30,1.88)
**Touch*Race**	Touch*Black	–	0.81 (0.64,1.02)	–	0.82 (0.61,1.11)	–	0.79 (0.58,1.07)	–	0.54 (0.25,1.18)
	Touch*White	–	Ref.	–	Ref.	–	Ref.	–	Ref.
	Touch*Other	–	0.86 (0.54,1.37)	–	0.86 (0.54,1.38)	–	1.58 (0.83,3.00)	–	1.31 (0.47,3.66)

Abbreviations: ITT, intention-to-treat; PP, per-protocol; IRR, incidence rate ratio; OR, odds ratio; SD, standard deviation; Ref., reference group.

[a]A comprehensive portal user was one who used eight or more full-tech functions (for full-tech participants).

[b]Includes African, American Indian or Alaska Native, Asian or Asian American, multiple races/ ethnicities, and unknown race/ethnicity.

© 2024 Walker DM et al. *JAMA Network Open.*

eTable 4: Sub-sample analysis of inpatient portal use outcomes for participants in the full-tech study arm per protocol, by touch status and race.

	Treatment							
	Touch		Race			Touch*Race		
	Low	High	Black	White	Other[a]	Touch*Black	Touch*White	Touch*Other
**Dining on Demand**								
Mean Prop, SD	0.22, 019	0.17, 0.13	0.21, 0.18	0.20, 0.17	0.24, 0.17	-	-	-
OR	Ref.	**0.64 (0.58,0.71)**	**1.28 (1.16,1.41)**	Ref.	**0.81 (0.69,0.96)**	0.95 (0.75,1.22)	Ref.	**1.67 (1.07,2.60)**
**Tutorial**								
Mean Prop, SD	0.19, 0.22	0.12, 0.10	0.20, 0.21	0.15, 0.17	0.15, 0.17	–	–	–
OR	Ref.	**0.48 (0.42,0.55)**	**1.59 (1.07,2.38)**	Ref.	0.71 (0.47,1.08)	1.16 (0.79,1.70)	Ref.	1.30 (0.60,2.84)
**To Learn**								
Mean Prop, SD	0.01, 0.05	0.03, 0.03	0.02, 0.05	0.02, 0.04	0.01, 0.02	–	–	–
OR	Ref.	**1.64 (1.38,1.95)**	1.32 (0.73,2.36)	Ref.	**0.37 (0.17,0.79)**	0.96 (0.35,2.67)	Ref.	**1.90 (1.07,3.37)**
**My Health**								
Mean Prop, SD	0.10, 0.17	0.13, 0.15	0.08, 0.13	0.12, 0.17	0.10, 0.16	–	–	–
OR	Ref.	**1.27 (1.07,1.51)**	0.80 (0.55,1.16)	Ref.	0.64 (0.41,1.00)	0.87 (0.71,1.06)	Ref.	1.03 (0.59,1.77)
**Happening Soon**								
Mean Prop, SD	0.37, 0.25	0.42, 0.19	0.38, 0.23	0.40, 0.23	0.38, 0.23	–	–	–
OR	Ref.	1.10 (0.97,1.25)	1.11 (0.86,1.44)	Ref.	**0.78 (0.73,0.82)**	0.86 (0.68,1.09)	Ref.	0.92 (0.78,1.07)
**Notes**								
Mean Prop, SD	0.00, 0.00	0.00, 0.00	0.00, 0.00	0.00, 0.00	0.00, 0.00	–	–	–
OR	Ref.	0.99 (0.35,2.79)	0.62 (0.16,2.42)	Ref.	**0.31 (0.15,0.62)**	1.97 (0.55,7.01)	Ref.	**5.52 (2.82,10.77)**
**Messages**								
Mean Prop, SD	0.03, 0.05	0.04, 0.03	0.03, 0.04	0.04, 0.04	0.03, 0.03	–	–	–
OR	Ref.	0.94 (0.80,1.10)	0.94 (0.47,1.87)	Ref.	0.51 (0.19,1.34)	1.41 (0.96,2.08)	Ref.	1.75 (0.64,4.78)
**I Would Like**								
Mean Prop, SD	0.00, 0.01	0.01, 0.01	0.00, 0.01	0.00, 0.01	0.00, 0.01	–	–	–
OR	Ref.	**2.42 (1.87,3.14)**	1.03 (0.30,3.49)	Ref.	**0.18 (0.06,0.52)**	1.06 (0.22,5.04)	Ref.	3.70 (0.38,35.97)
**My Chart**								
Mean Prop, SD	0.03, 0.04	0.03, 0.03	0.03, 0.04	0.03, 0.03	0.04, 0.05	–	–	–
OR	Ref.	1.09 (0.85,1.38)	1.05 (0.57,1.91)	Ref.	0.62 (0.21,1.82)	1.28 (0.79,2.08)	Ref.	2.38 (0.79,7.13)
**Taking Care of Me**								
Mean Prop, SD	0.04, 0.05	0.05, 0.03	0.04, 0.04	0.05, 0.05	0.04, 0.03	–	–	–
OR	Ref.	1.00 (0.86,1.15)	0.75 (0.51,1.11)	Ref.	0.63 (0.29,1.35)	**1.59 (1.24,2.05)**	Ref.	1.12 (0.50,2.53)

Abbreviations: OR, odds ratio; Prop, proportion; SD, standard deviation; Ref., reference group.

© 2024 Walker DM et al. *JAMA Network Open.*

[a]Other includes African, American Indian or Alaska Native, Asian or Asian American, multiple races/ethnicities, and unknown race/ethnicity.

© 2024 Walker DM et al. *JAMA Network Open.*
