# Supplementary material for: Differences by Race in Outcomes of an In-Person Training Intervention on Use of an Inpatient Portal: A Secondary Analysis of a Randomized Clinical Trial
Source: JAMA Netw Open. 2024 Apr 4;7(4):e245091. doi: 10.1001/jamanetworkopen.2024.5091 (PMC11192182; doi:10.1001/jamanetworkopen.2024.5091)
Supplement: Supplement 2. — eAppendix 1. Trial Protocol eAppendix 2. Original Protocol [file jamanetwopen-e245091-s002.pdf]

eAppendix 1. Trial Protocol

High Tech and High Touch (HT2): Transforming patient engagement throughout the continuum of care by engaging patients with portal technology at the bedside

NCT02943109

Study Protocol

Prepared By:

Ann Scheck McAlearney, ScD, MS[1,2,3,4]; Daniel M. Walker, PhD, MPH[1,2]; Naleef Fareed, PhD, MBA[2,3]; Lindsey Sova, MPH[2]; Cynthia J. Sieck, PhD, MPH[1,2]; Timothy R. Huerta, PhD, MS[1,2,3,4]

[1] Department of Family and Community Medicine, College of Medicine, The Ohio State University.
[2] CATALYST, Center for the Advancement of Team Science, Analytics, and Systems Thinking in Health Services and Implementation Science Research, College of Medicine, The Ohio State University.
[3] Department of Biomedical Informatics, College of Medicine, The Ohio State University.
[4] Division of Health Services Management and Policy, College of Public Health, The Ohio State University.

Version 2.0 (December 12, 2019):	Final pre-analysis Protocol
Version 2.1 (March 16, 2021):	Updated to reflect request from external reviewer to include sensitivity analysis.
Version 2.2 (November 7, 2023):	Updated to correct randomization and factor protocol deviations within analytic populations.

Table of Contents

Section 1: Administrative information 4

1.1. Clinical trials registration 4

1.2 Ethics approval 4

1.3 Availability of data 4

1.4 Competing interests 4

1.5 Funding 4

1.6 Roles and responsibilities 4

1.7 Acknowledgements 4

1.8 Protocol revisions 5

Section 2: Introduction 9

2.1 Background and rationale 9

2.2 Objectives 9

Section 3: Study methods 11

3.1 Trial design 11

3.2 Randomization 14

3.3 Blinding 14

3.4 Sample size 15

3.5 Statistical interim analysis and stopping guidance 15

3.6 Timing of final analysis 15

3.7 Study flow 15

3.8 Timing and mode of outcome assessment 16

Section 4: Statistical principles 18

4.1 Confidence intervals and p-values 18

4.2 Adherence and protocol deviations 18

4.3 Analysis populations 19

Section 5: Trial population 20

5.1 Screening and eligibility 20

5.2 Recruitment 21

5.3 Study withdrawal 22

5.4 Baseline patient characteristics 22

Section 6: Data management 23

6.1 Data sources 23
6.2 Data model 23
6.3 Data processing 23
Section 7: References 28

Section 1: Administrative information

1.1. Clinical trials registration
This study is registered with clinicaltrials.gov: NCT02943109.

1.2 Ethics approval
The Institutional Review Board of The Ohio State University (OSU), College of Medicine (COM) approved the study.

1.3 Availability of data
The data used in this study includes identifiable information, and per our consent approval we are not permitted to share study data. However, the data management protocol and analytic approach will be made publicly available via publication.

1.4 Competing interests
The authors declare that they have no competing interests to report.

1.5 Funding
This work was supported by the Agency for Healthcare Research and Quality (AHRQ) Grants R01HS024091, R21HS024767, P30HS024379. While this research was funded by AHRQ, the study sponsor had no involvement in the collection, analysis, or interpretation of data, in the writing of this article, or in the decision to submit the article for publication.

1.6 Roles and responsibilities
ASM, TH, JH, CS, DW, AA, LS, MR, SMB designed the study, and with NF, SS, GD, SRM, and AG developed and approved the initially submitted version of this manuscript. DW and LS wrote the Protocol, and TH was the senior statistician responsible for the Protocol. ASM was the Principal Investigator of the study. All authors read and approved the final manuscript.

1.7 Acknowledgements
The authors would like to thank the following individuals who were affiliated with the authors' organization during the project for their assistance:
Technical Support: Robert Taylor, Lakshmi Gupta
Research Associates: Ayanna Scott, Jaclyn Volney, Toby Weinert, Alison Silverman, Danijela Cvijetinovic, Karen Alexander, Kimberly Karels
Administrative: Pamela Thompson

1.8 Protocol revisions

Revision history of the protocol, including rationale, is provided in Table 1, sorted by date.

Table 1: Amendments to the study protocol (sorted by date).

Date / Section reference	Revision	Rationale
**February 2016**<br>**Section 3.1**	**Low-Tech intervention:** Changed from no tablet to Lite-Tech intervention with a tablet with a limited MyChart Bedside feature set.	Independent of the study, OSUWMC decided to implement MyChart Bedside system-wide, resulting in it not being possible to not provide some patients a tablet.
**February 2016**<br>**Section 3.4**	**Study site:** Changed study sites from six matched unit pairs to OSUWMC system-wide, including six hospitals.	Independent of the study, OSUWMC decided to implement MyChart Bedside system-wide, resulting in it being feasible to expand the study sites across the OSUWMC.
**February 2016**<br>**Section 5.1**	**Focus on multiple chronic conditions:** Changed from inclusion criteria of two or more chronic conditions to no disease restrictions.	Independent of the study, OSUWMC decided to implement MyChart Bedside system-wide, resulting in it being infeasible to restrict the sample based on disease characteristics.
**February 2016**<br>**Section 5.1**	**Inclusion criteria:** Criteria of patient admitted within last 24 hours and expected discharge in more than 72 hours removed.	The expansion of the study from the six matched unit pairs to OSUWMC system-wide resulted in logistic challenges in being able to reaching all patients quickly. Additionally, OSUWMC does not store expected discharge in its Epic Clarity tables, making this criterion impossible to operationalize.
**February 2016**	**Low-Touch intervention:** Changed from active control where patients receive an in-person visit from a Technology Navigator (TN) to a passive control where patients were directed to self-guided learning materials built into MyChart Bedside.	Independent of the study, OSUWMC decided to implement MyChart Bedside system-wide. This change increased the number of potentially eligible patients for TNs to visit, presenting a logistical challenge. Additionally, in the original protocol where patients assigned to the Low-

**Section 3.1**		Tech arm did not receive a tablet, it was not possible to deliver a passive control. However, when the protocol changed to all study participants receiving a Tablet (Full/Lite-Tech), a passive control arm was possible. This approach resolved the logistical challenges.
**December 2016**<br>**Section 2.2**	**Hypotheses:**<br>The primary and secondary hypotheses were revised and reduced from seven to four hypotheses.	In the original proposed study, the plan was to execute it using 12 units and limit the study to chronic diseases. Additionally, participants were to be randomized to receive no technology or a full version of the MyChart Bedside application on the tablet. OSUWMC made several decisions that had an effect on the study design. In particular, OSUWMC determined that not providing a tablet was no longer an option; however, they made the units across the entire hospital system available for the study. The research team worked with OSUWMC to refine the hypotheses needed to reflect the more inclusive study. This resulted in dropping readmissions and comfort with technology as outcomes of interest, among other revisions.
**July 2017**<br>**Section 4.2.1**	**Improve enrollment yield:**<br>A research team member may visit the patient to remind them to complete the survey, limited to three visits per patient, once per day. Patients without completed surveys could also be visited on subsequent hospital admissions to complete their survey.	Quality assurance testing of the data collection system identified a technical problem which caused an unusual pattern of missing responses. Inspection of completeness of questions identified a pattern of non-completion such that every 5-10 questions, a question would demonstrate a considerably lower percent missing than the questions asked immediately before and after it. This pattern was attributed to the display of the survey on Qualtrics that hid some questions from view. A reminder was added to the survey to inform respondents if they had left questions unanswered as they clicked the button to advance

		the survey, still allowing them to advance if that was their preference.
**July 2017**<br><br>**Section 4.2.1**	**Study enrollment:**<br>A confirmation was added to each page of the survey to inform respondents when they had left questions unanswered on a page. They could continue to advance if that was their preference.	Completion of the survey was found to limit the number of patients in the High-Touch study arm. This change represented a deviation from the original protocol which had required that the Admission survey be completed prior to intervention delivery.
**February 2018**<br><br>**Section 3.1.3**	**Study allocation:**<br>The allocation of individuals in arms were rebalanced to ensure sufficient numbers of participants in the High-Touch treatment arm due to a significant number of patients becoming lost to follow-up.	Quality monitoring of the study found a number of exogenous factors that were impacting the High-Touch intervention, including, but not limited to: patient discharges; patient moves to other rooms; patients not present during rounding; patients' engagement with the care team; and tablet returns prior to receiving an intervention. This change was done to ensure that sufficient individuals were assigned to the High-Touch intervention to increase the number of patients who would be eligible to receive a High-Touch intervention.
**March 2018**<br><br>**Section 4.2.1**	**Intervention available after consent:**<br>TNs are allowed to make first contact for the High Touch intervention once the patient consented – no longer waiting for a complete Admission Survey. The TN should work with the patient to walk them through completing the survey during the intervention.	As noted in Section 3.1.3 changes were made to the intervention protocol to facilitate greater engagement in the High-Touch arm. Specifically, prior to February 2018, participants assigned to the High-Touch intervention group did not receive the intervention until after they had completed the Admission survey. Starting on February 22, 2018, the research team modified the protocol to allow TNs to mark a survey as complete and allow the intervention team to perform the High-Touch intervention. In March 2018, 22 days later, the protocol was once again changed to deliver the High-Touch intervention at first contact with the TN if they had been assigned to the High-Touch intervention.

**July 2018**<br><br>**Section 4.2.1**	**Intervention assignment:** Individuals that had activated a MyChart Bedside account prior to the study start or in a Full-Tech unit were manually assigned to the Full-Tech intervention regardless of their MRN. Individuals who were found to have accessed both Full and Lite versions over the course of the study were excluded from the analysis for this study.	Quality assurance testing of ongoing data collection of the MyChart Bedside usage data revealed that patients who were to have been assigned to Lite-Tech based on their MRN were, in fact, using functions only available on Full-Tech. Subsequent review also found the reverse to be true, participants that were assigned to Full-Tech were limiting their usage to those functions in Lite-Tech.<br><br>The study team conferred with Epic–the electronic health record (EHR) vendor that developed the code to assign patients to a particular arm of Tech status–and manually reconstructed Tech assignment using the original assignment code. A cohort of individuals were identified as not having received the intended Tech assignment. Through this process, the study team was able to secure the true Tech intervention status. Individuals that had activated a MyChart Bedside account or were in a Full-Tech unit prior to the study start were manually assigned to Full-Tech regardless of their MRN. Additionally, individuals who were found to have accessed both Full and Lite versions over the course of the study were identified and subsequently excluded from the analysis for this study. This scenario may have occurred when a patient was assigned an MRN at admission but subsequent records review identified an existing MRN. In these cases, individuals unexpectedly transitioned between two Tech intervention states.

Section 2: Introduction

2.1 Background and rationale

For patients with complex care needs, engagement in disease management activities is particularly critical.[1-4] The human cost of chronic illnesses touches almost every person in the US. These costs are real, personal and pervasive.[5] In response, patients often seek out tools to help them manage their health. Patient portals–a Personal Health Record (PHR) tethered to an Electronic Health Record (EHR)–show promise as tools that patients accept and value and that can improve health.[6-10] Although patient portals currently focus on the outpatient experience, the Ohio State University Wexner Medical Center (OSUWMC) has deployed a portal designed specifically for the inpatient experience that is connected to the ambulatory patient portal available after discharge. While this inpatient technology is in active use at only one other hospital in the US, healthcare facilities are currently investing in the infrastructure necessary to support large-scale deployment.[11]

Times of acute crisis such as hospitalization may increase a patient's focus on his/her health, a factor that has been linked in other areas to a greater awareness of health risks and increased focus on health behaviors.[12-14] During this time, patients may be more engaged with their care and therefore particularly interested in the potential for using tools to manage their health after discharge. Evidence shows that enhanced patient self-management can lead to better control of chronic illness,[1,15,16] and patient portals may serve as a mechanism to facilitate increased engagement.[17-19]

Our proposed four-year study used a mixed-methods approach to evaluate a pragmatic randomized controlled trial (RCT) studying the effectiveness of a High-Tech Intervention (MyChart Bedside, the inpatient portal), and an accompanying High-Touch Intervention (training patients to use the portal to manage their care and conditions) in a sample of hospitalized patients. This study measured how a patient portal tailored to the inpatient stay could improve patient experience and increase patient engagement by 1) improving patients' perceptions of the process of care while in the hospital, 2) increasing patients' self-efficacy for managing their health and health care, and 3) facilitating continued use of a patient portal for care management activities after discharge. In addition, we aimed to enhance patients' use of the patient portal available to outpatients, MyChart, once they were discharged.

2.2 Objectives

The ***High Tech and High Touch (HT2): Transforming patient engagement throughout the continuum of care by engaging patients with portal technology at the bedside*** study at OSU Wexner Medical Center (OSUWMC) sought to study how the deployment of a commercial (i.e., Epic Systems) inpatient portal, MyChart Bedside, impacted identified patient outcomes. To evaluate the impact of function set and training

on inpatient (i.e., MyChart Bedside) and outpatient (i.e., MyChart) portal use, the HT2 Study[20] was designed as a large-scale, pragmatic randomized controlled trial across six hospitals in a healthcare system.

Using a two-by-two factorial design, HT2 simultaneously examined the effects of two approaches to portal training with two versions of the inpatient portal. HT2 was designed to test four study hypotheses:

Hypotheses related to primary outcomes:

H1: Patients assigned to the High intervention levels for Tech and Touch will demonstrate higher use of MyChart Bedside.

H2: Patients assigned to the High intervention levels for Tech and Touch will demonstrate higher levels of satisfaction with (H2a) and involvement in (H2b) their care experience.

Hypotheses related to secondary outcomes:

H3: Patients assigned to the High intervention levels for Tech and Touch will demonstrate higher rates of outpatient portal adoption (i.e., of Epic's MyChart) for those admitted without prior outpatient portal use (H3a), and higher use of MyChart for those admitted who had previously used the outpatient portal (H3b).

H4: Patients assigned to the High intervention levels for Tech and Touch will demonstrate higher levels of self-efficacy.

To prevent outcome reporting bias and data-driven analysis of results, we produced this detailed statistical analysis plan. The plan was originally developed and drafted prior to examination of the data, and in the absence of randomization knowledge. The plan has been revised as the analysis has proceeded.

Section 3: Study methods

3.1 Trial design

This study was designed as a pragmatic factorial randomized controlled trial of two concurrent patient portal-related interventions.

3.1.1 Intervention description: Tech - Full/Lite

The Full-Tech intervention consisted of the standard build of the MyChart Bedside product as delivered from Epic and augmented by OSUWMC at the start of the study. The application was served by a *Home* landing page that displays events scheduled for the day and recent health updates and included access to 10 additional functions located in tabs along the side of the inpatient portal image:

1. *Home*: presented events scheduled for the day and data on patient vitals, including last blood pressure reading, current pulse and other patient vital signs refreshed from the Electronic Health Record (EHR) once every 5 minutes[i]
2. *Bedside Tutorial*: tutorial materials for the use of MyChart Bedside, including an 11-minute video explaining how to use MyChart Bedside and access to PDF versions of tutorial materials
3. *Dining on Demand*: food ordering service
4. *To Learn*: educational resources for the patient based on their treatment plan
5. *Happening Soon*: a timeline for the care activities that the patient could expect for the day
6. *Taking Care of Me*: information detailing members of the patient's care team
7. *Messages*: non-urgent secure messaging with the patient's care team
8. *My Health*: recent vital signs and lab test results
9. *Notes*: write/record reminders and notes
10. *I Would Like*: non-urgent requests from pastoral care or gift shop
11. *OSU MyChart*: allowed the patient to access MyChart, including creating a new account

In addition to the *Home* landing page, the Lite-Tech version of MyChart Bedside consisted of the 3 functions: *Bedside Tutorial*, *Dining on Demand*, and *To Learn*.

3.1.2 Intervention description: Touch - High/Low

The education and training intervention, referred to as Touch, was performed by trained Technology Navigators (TNs) who deliver training tailored to both the version of

[i] This function was automatic, so was not considered selectable and not included in the count of functions for either the Full- or Lite-Tech versions.

MyChart Bedside installed on their tablet (both Full- and Lite-Tech) and the perceived capacity of the patient.

Patients assigned to the Low-Touch intervention received an intervention focused on providing them with resources to conduct self-guided learning of MyChart Bedside. Specifically, TNs directed patients to the tutorial videos integrated in the MyChart Bedside application that explained the portal's functions.

In contrast, patients assigned to the High-Touch intervention would systematically review each of the functions of MyChart Bedside with the TN who would discuss how and when they are appropriately used. Patients were encouraged to use their tablet and follow along with the training as the TN moved through each function and ask the TN questions about their records during the presentation. The TNs tailored their presentation based on a patient's demonstrated knowledge of the technology to minimize patient participation burden while focusing on explaining the value of each function within the application. Patients unavailable after three intervention attempts were converted to Low-Touch.

3.1.3 Allocation ratio

The Tech intervention contrasted two function sets–Lite-Tech (40% allocation), which was a minimalistic toolset focused on providing basic data to the patient, and Full-Tech (60% allocation), a more expansive toolset. The Touch intervention contrasted two training approaches–Low-Touch, a self-guided approach where patients could view built-in tutorials, and High-Touch (initially 50% allocation), where patients receive in-person guided training with the technology. The realized allocation ratios are presented in Table 2.

Table 2. Realized allocation ratio.

Tech Allocation	Touch Allocation		
	Low	High	Total
Lite			
Frequency	275	470	745
Row %	36.91%	63.09%	100.00%
Column %	23.31%	27.45%	25.76%
Overall %	9.51%	16.25%	25.76%
Full			
Frequency	905	1,242	2,147
Row %	42.15%	57.85%	100.00%
Column %	76.69%	72.55%	74.24%
Overall %	31.29%	42.95%	74.24%
Total			
Frequency	1,180	1,712	2,892
Row %	40.80%	59.20%	100.0%
Column %	100.00%	100.00%	100.0%
Overall %	40.80%	59.20%	100.0%

The assignment protocol used three phases where the Touch allocation ratio was changed by the research team.

1. In Phase 1, patients received the High-Touch intervention only after consenting to the study and submitting their Admission survey (see Section 3.8.2). Low-Touch patients could view the tutorial at any point and were not required to complete the Admission survey. Phase 1 assigned patients to High/Low-Touch in a 1:1 ratio.
2. In Phase 2, beginning in February 2018, the study shifted to a 3:1 ratio of High/Low-Touch. This change was done to ensure that sufficient individuals were assigned to the High-Touch intervention due to under-enrollment. Quality monitoring of the study found a number of exogenous factors that were impacting the High-Touch intervention, including, but not limited to: patient discharges; patient moved to other rooms; patient not present during TN rounding; patient was engaged with the care team and unable to work with the TN; and tablet returned prior to receiving an intervention. This change in the ratio was intended to increase the number of patients who would be eligible to receive a High-Touch intervention while maintaining the *per protocol* approach in that these exceptions were considered a component of the Protocol.
3. Phase 3 began in March 2018 and lasted through the end of the study in September, 2018. This phase maintained the 3:1 split, but removed the requirement that patients complete the Admission survey prior to receiving the High-Touch intervention. Completion of the survey was found to limit the number of patients in the High-Touch study arm. This change represented a deviation

from the original protocol which had required that the Admission survey be completed prior to intervention delivery (see Section 4.2.1).

3.2 Randomization

Participants were randomized into each arm of each intervention, Tech and Touch, in separate processes.

3.2.1 Tech randomization

Tech randomization was accomplished using a 4-digit random number table generated using Stata version 14.0 which was used as a comparison table against the last four digits of the patient's Medical Record Number (MRN) which was assigned prior to study assignment. A preliminary simulation analysis found that random assignment using this model created appropriately sized groups of 40% and 60%.

Individuals who had access to the Full version of MyChart Bedside prior to enrollment in the study (i.e., those patients who were admitted to the Cancer Center, which was not included as a site, or patients who had used MyChart Bedside during the pilot phase of implementation in which only Full-Tech was available) maintained their Full-Tech status and were excluded from randomization if they were transferred to a unit included in the study.

3.2.2 Touch randomization

The study protocol included a series of robust approaches to randomization. Generally, Touch randomization occurred at consent using the randomizer function in Qualtrics which was used for both consent and survey data collection. The Protocol included a procedure for the assignment of Touch in cases where the patient was checked in after daily census and was not included in the Qualtrics randomization (i.e., due to timing of reconciliation between report pulls and the start of the work day). In those cases, a predefined randomization table was used to assign Touch status.

3.3 Blinding

Both the standard and alternate processes for Touch assignment occurred prior to study consent and therefore the TN, the patient, and the care team were blind to their study assignment at consent. Additionally, at the time of provisioning, both the patient and the nurse that assigned the patient the tablet were blind to Tech assignment. TNs providing the intervention were blind to Tech assignment prior to consent, but not prior to study eligibility assessment.

3.4 Sample size

The study did not adopt a sampling frame and represented a census approach to the implementation. The Consort Diagram (see Figure 1), provides information on the pragmatic factors that limited enrollment.

The original protocol calculated power based on 2016 Hospital Consumer Assessment of Healthcare Provider and Systems (HCAHPS) patient satisfaction scores as a baseline for outcome measures using G*Power 3.1 software. An expected enrollment of 3,872 was planned with the design expected to be sensitive enough to identify changes of between 2.2% and 3.8% across the panel of HCAHPS patient satisfaction scores.

3.5 Statistical interim analysis and stopping guidance

No interim analyses were planned or conducted for our study. Since HT2 posed minimal risk, stopping guidance was not applicable.

3.6 Timing of final analysis

HT2 was a minimal risk study, and as a result, final outcomes and their association with the interventions were reviewed only upon study completion.

3.7 Study flow

Patient participation in the study followed the CONSORT diagram shown in Figure 1.

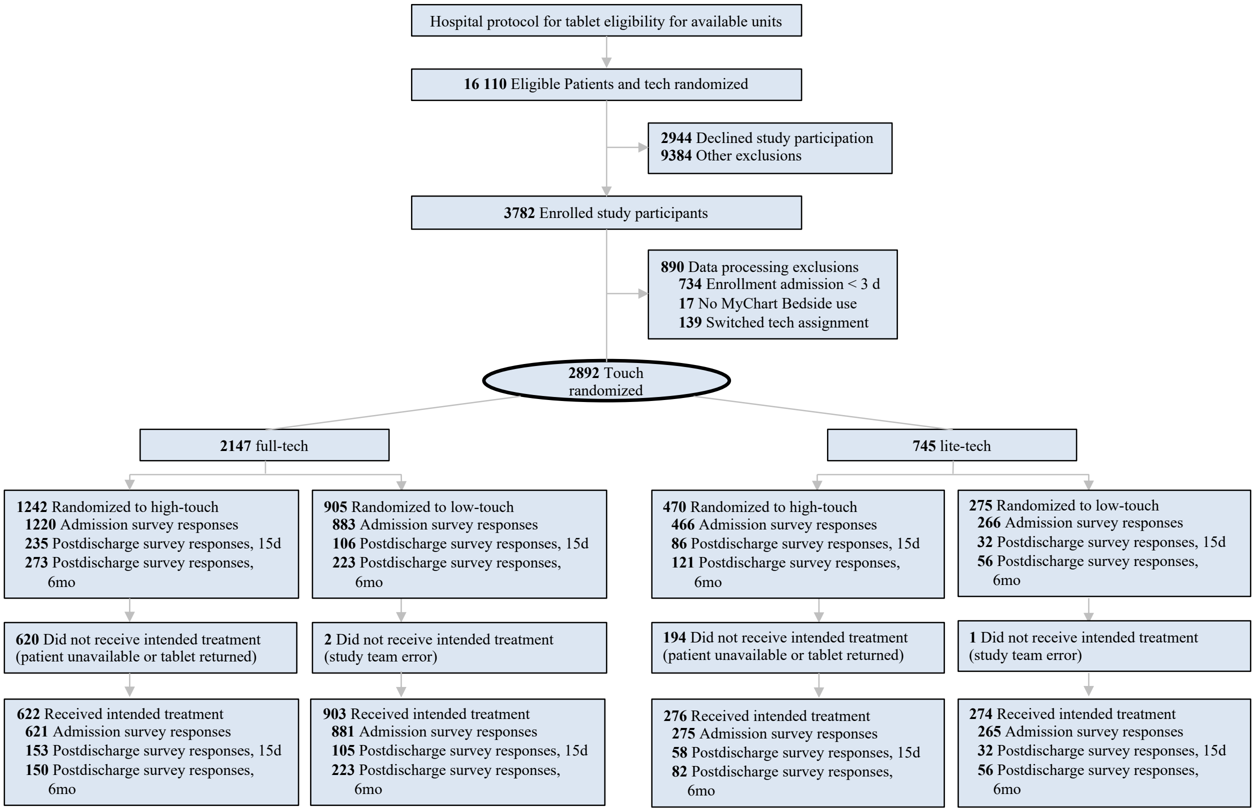

Figure 1. CONSORT diagram.

3.8 Timing and mode of outcome assessment

The outcomes for this study included measures of use of both the inpatient and outpatient portal as well as measures of satisfaction and self-efficacy.

3.8.1 Portal usage

MyChart Bedside and MyChart usage was determined through the use of log file analysis (LFA). The methods associated with LFA have been extensively discussed in methods papers published by the research team. Log files provided the date, time, and type of interaction with each of the functions in both of the patient portals. The log file data that was available included:

1. Login date and time
2. Functions accessed
3. Activity notes

Log files were linked to the patient MRN within the Epic Clarity clinical data record. MyChart Bedside and MyChart log files were obtained in an identified form for study

participants for purposes of matching this data to Admission and Post-discharge surveys and follow-up interviews (see Section 6 - Data Management).

3.8.2 Satisfaction and self-efficacy

Data on satisfaction and self-efficacy were collected via survey at enrollment (referred to as the Admission survey), between 10 and 21 days post-discharge (referred to as the 15-day Post-discharge survey) and, finally, at six months post-discharge (referred to as the 6-month Post-discharge survey). The three surveys each gathered data on the outcomes to allow for comparisons at different time points.

The Admission survey included questions related to self-efficacy, hospital satisfaction, and patient experience. The Admission survey was administered in two modes: a paper form and electronically. The electronic survey was created and collected using Qualtrics online survey software. Patients accessed the survey via the MyChart Bedside portal. The default administration mode was electronic; paper forms were only given if the patient requested it or encountered a technical issue with the hospital-issued tablet that affected their ability to complete the survey electronically.

3.8.3 Post-discharge survey administration

Both the 15-day and 6-month Post-discharge surveys were administered based on the communication preference of the participant. During the consent process, participants were asked to identify a communication preference–email or phone–through which they would be contacted for the follow-up surveys. The study team developed a robust communication approach that would initiate contact based on stated preference, allow for participants to change their preference throughout the study, and would leverage alternate approaches if the participant could not be reached using their preferred approach. When email was used, the research team would send the participant an invitation and a link to the Qualtrics online survey and up to three reminders. When a phone call was used, the research team would attempt up to three calls. Communication efforts for Post-discharge surveys began 10 days post-discharge from the enrollment admission and 24 weeks post-discharge from the enrollment admission.

Section 4: Statistical principles

4.1 Confidence intervals and p-values

For all statistical tests, a p-value of 0.05 was used to assess significance and 95% confidence intervals were reported. No adjustments were made for multiple hypothesis testing.

4.2 Adherence and protocol deviations

Protocol deviations may have occurred if:

1. a participant consented to participate in the study but subsequently requested a change in Tech status to the OSUWMC;
2. a participant was admitted to the OSUWMC, was assigned to the Lite intervention, and was subsequently admitted to a unit that always received the Full version of MyChart Bedside.
3. a participant did not receive the High Touch intervention as a result of being unavailable during multiple attempts by the TN (e.g., care team was present).

In cases (1) and (2) above, the individual was removed from the study. In case (3), the individual was retained in our analytic sample and considered according to the potential protocol deviations as detailed in the Statistical Analytic Plan Section 1.4.1.

4.2.1 Operational protocol amendments

<u>Operational protocol amendment 1: Improve enrollment yield (Jan 2017)</u>

A research team member could visit the patient to remind them to complete the survey, limited to three visits per patient, once per day. Patients without completed surveys could also be visited on subsequent hospital admissions to complete their survey.

<u>Operational protocol amendment 2: Improve data quality (Jul 2017)</u>

A confirmation was added to each page of the survey to inform respondents when they had left questions unanswered on a page. They could continue to advance if that was their preference.

<u>Operational protocol amendment 3: Intervention available after consent (Feb 2018)</u>

TNs were allowed to make first contact for the High-Touch intervention once the patient consented–no longer waiting for a complete Admission survey. The TN should work with the patient to walk them through completing the survey during the intervention.

<u>Operational protocol amendment 4: Intervention assignment (Mar 2018)</u>

Individuals that had activated a MyChart Bedside account or were in a Full-Tech unit prior to the study start were manually assigned Full-Tech regardless of their MRN.

Individuals who were found to have accessed both Full and Lite versions over the course of the study were to be excluded from the analysis for this study.

4.3 Analysis populations

Estimates of the intervention effects were calculated on two analytical populations. The primary analytic population of the study used an *intention-to-treat (ITT)* approach. Using this paradigm, study participants assigned to the High-Touch group that ended up not receiving the High-Touch intervention and patients (n = 3) assigned to the Low-Touch group but received the training intervention due to patient request, were analyzed on the basis of their randomized study group.

The effects of the intervention were additionally evaluated on a *per-protocol* population, composed exclusively by those participants who were administered their randomized intervention.

Table 3. *Per-protocol* population's allocation ratio.

**Tech Allocation**	**Touch Allocation**		
	Low	High	Total
Lite			
Frequency	275	276	550
Row %	49.82%	50.18%	100.00%
Column %	23.28%	30.73%	26.51%
Overall %	13.20%	13.30%	26.51%
Full			
Frequency	903	622	1,525
Row %	59.21%	40.79%	100.00%
Column %	76.72%	69.27%	73.49%
Overall %	43.52%	29.98%	73.49%
Total			
Frequency	1,177	898	2,075
Row %	56.72%	43.28%	100.0%
Column %	100.00%	100.00%	100.0%
Overall %	56.72%	43.28%	100.0%

Section 5: Trial population

5.1 Screening and eligibility

All admitted patients were screened at admission by nursing staff for eligibility to use a standardized tablet and the associated MyChart Bedside application based on the following protocol, developed by OSUWMC:

1. Patients must have been admitted to a hospital in the OSUWMC system where MyChart Bedside has been deployed.
2. Patients must have been capable of accepting the tablet terms of service.
3. Patients must not have had an excluded status, which were:
    a. less than 18 years old;
    b. legally blind;
    c. cannot speak and/or read English;
    d. involuntarily confined or detained; or,
    e. be considered as having a diminished decision-making capacity.

Nursing staff used their judgement in terms of whether a patient may have had an additional reason for exclusion and documented those reasons in the EHR. Acceptable reasons for exclusion included that the patient declined to use a tablet or the patient was not coherent at the time the nursing staff was rounding for the purpose of providing tablets. Patients were provided Android tablets by the medical center for use during their stay. The provisioning process defined the patient population and was outside of the scope of the study.

5.1.1 Inclusion criteria

Study inclusion criteria were as follows:

1. patient accepted tablet and use of MyChart Bedside;
2. patient was available in their room; and,
3. patient was capable of informed consent.

Only patients who were provisioned with MyChart Bedside were included in the study.

5.1.2 Exclusion criteria

Study exclusion criteria were as follows:

1. the patient was less than 18 years of age.

The inclusion criteria, by design, means that family and proxy users were excluded from the study.

5.2 Recruitment

The AMC Epic Clarity tables were used to develop a fully identified custom Census Report which provided information on all patients who met the inclusion criteria present in the hospital at midnight of each day. The research team integrated this report with the study database to create a daily list of individuals that had consented, refused, not been provided a tablet, or were eligible for being visited by a TN during TN rounding. This information was used to set up a rounding schedule to visit the rooms of these patients to follow up about study participation as follows:

1. In cases where a patient had not been provisioned and there were no clinical notes detailing a rationale for the withholding of access, members of the research team would approach the nursing team and ask them to make a formal determination about the appropriateness of the tablet for the patient.
2. In cases where a patient was provisioned, the study team would put the patient on the rounding schedule and attempt to recruit them to the study.

5.2.1 Study enrollment, participation, and remuneration

Eligible identified patients were invited to participate in the study via a link in MyChart Bedside which presented an electronic combined HIPAA authorization and consent form approved by the AMC's Institutional Review Board (IRB) for patients to sign to authorize extraction of identified clinical data. Study staff approached eligible patients to identify their interest in participation. Interested patients were guided to the tab within MyChart Bedside that explained that participation included the following tasks and consent:

1. a survey to be taken immediately upon completing consent (the Admission survey);
2. two surveys that would be taken by phone or via an emailed link;
    a. The 15-day Post-discharge survey
    b. The 6-month Post-discharge survey
3. additionally, patients were notified that they may be contacted for up to two telephone interviews: within 21 days of discharge and at approximately 6 months post-discharge. Not all individuals would be invited to participate in these interviews;
4. consent to share their clinical data, including the log files associated with their use of patient portals.

Upon completion of each survey, participants had the option to enter contact information on a separate page for a raffle of a $100 gift card and for future follow-up for additional surveys and interviews. One gift card was raffled off each week throughout data collection.

Participants were provided the option to request a paper combined HIPAA authorization and consent form to sign, delivered by a study team member. Participants received an electronic copy of the signed combined HIPAA authorization and consent form or a paper version from a study team member within 24 hours. If the patient had been discharged, a paper version was mailed to the address on file.

5.3 Study withdrawal

All data collected during the period as study participants was included in the analysis. Individuals who chose to withdraw were, in effect, refusing to contribute further data, including the following:

1. Patients who removed themselves from the study prior to discharge in the admission in which they are enrolled had no data about them included in the study.
2. Patients who removed themselves from the study after discharge in the admission in which they are enrolled had the Admission survey data and MyChart Bedside use data about them included in the study, but depending on when they withdrew, may not have any data about them included as part of either of the Post-discharge surveys.
3. Patients who were not reachable after discharge for the 15-day Post-discharge survey were not considered lost to follow-up, and contact was attempted for the 6-month Post-discharge survey.

5.4 Baseline patient characteristics

Baseline characteristics were summarized, including age (median), gender (male, female percentages), race (White, Black, Other percentages), length of stay (days), and the Charlson comorbidity index (median, treated as a continuous score). Baseline characteristics were compared using analysis of variance (ANOVA) or t-tests, as appropriate, to examine equivalence between the four study arms.

Section 6: Data management

6.1 Data sources

Data for this study was merged from two sources–one delivered from surveys promulgated by Qualtrics, a cloud-based survey system, and the other from the OSUWMC Epic Clarity tables. Data was kept in separate tables in accordance with the study data model.

6.2 Data model

Five tables were used in the data model used for this study. Figure 2 illustrates the entity data diagram for each table in the data model and their relationship.

Data Table 1: **Demographic Data**	Data Table 2: **Survey & Intervention Data**	Data Table 3: **Encounter Data**	Data Table 4: **Inpatient Portal Use Data**	Data Table 5: **Outpatient Portal Use Data**
**Record Identifier:** ParticipantID	**Record Identifier:** ParticipantID	**Record Identifier:** ParticipantID EncounterID	**Record Identifier:** ParticipantID EncounterID	**Record Identifier:** ParticipantID
**Provenance:** Epic Clarity	**Provenance:** Qualtrics	**Provenance:** Epic Clarity	**Provenance:** Epic Clarity	**Provenance:** Epic Clarity
**Description:** This dataset contains demographic information about each study participant.	**Description:** This dataset contains survey responses for the three study surveys as well as data about the intervention.	**Description:** This dataset contains information about each study participant's inpatient hospital encounter at an AMC facility.	**Description:** This dataset contains processed summaries of MyChart Bedside use at the session level for study participants.	**Description:** This dataset contains processed summaries of MyChart outpatient portal use at the session level for study participants.

Figure 2. Entity data diagram.

Data Tables 1 and 2 provided data related to the participant. Intervention data was written into Qualtrics records from the EHR via a passthrough using a secure application programming interface (API). Data Tables 3 and 4 provided data about the patient's encounter history during the study period as well as their use of the inpatient portal for each visit. Data Table 5 provided data about the use of the outpatient portal, which therefore meant it was not related to a specific encounter. Where possible, the original data in the associated Epic Clarity tables were delivered to the research team, including the Admission/Discharge/Transfer (ADT) table. This approach was used to ensure that joins were completed by the research team as opposed to an intermediate data broker and ensure data quality.

6.3 Data processing

As noted in Section 6.2, data from the original Epic Clarity tables were provided to the research team. This process was deemed necessary, as quality testing of the data found a number of join errors that created issues when using the study and EHR

derived data. We detailed ten specific data quality issues that were found and resolved in the data quality assurance testing.

6.3.1 Multiple MRNs

MRNs were used as primary identifiers by both the hospital and in the context of the study. Moreover, MRNs were used throughout data processing to associate data with a unique patient. Typically, each unique patient had a single, unique MRN. In rare cases, for instance when a patient presented in the emergency room and was unable to provide identification, a patient may have been temporarily issued an MRN. While hospital records are adjusted to ensure a single, unique MRN at a later time, data was written to the log files with the temporary MRN. Patients with multiple MRNs were identified, along with their duplicate MRNs, and the identifiers were recoded to a “primary” MRN in the processed data.

6.3.2 Combining hospital encounters

In preliminary data processing, it was discovered that the ADT report contained both overlapping admissions as well as two separate admissions within close time proximity. These scenarios commonly occurred when an individual moved facilities for diagnosis and treatment; for instance, in the case of an individual in the general hospital moving to the heart hospital for testing. To resolve these issues, data analysts merged admissions within 4 hours of each other, as well as overlapping admissions, and considered them a single admission. Data quality tests found that approximately 9% of discharge-subsequent admission and overlaps were resolved by this threshold. In cases where a discharge was recorded and a subsequent admission was recorded more than four hours later, that admission was deemed to be a new, separate admission.

6.3.3 Matching admission surveys to a patient encounter

Both the MRN and the date and time of the start of the Admission survey was used to match an Admission survey to a specific patient encounter in the ADT table. However, Admission surveys were collected using two modalities–electronic and paper–the latter of which did not produce an electronic timestamp for survey start. In the first case, electronic data capture via the Qualtrics-hosted survey, MRN, and survey start/stop times were written to Data Table 2, however in cases where paper surveys were used, no such recording of start/ stop time was automatically recorded. In this latter case, the date and estimated time the survey was delivered was recorded in a separate administrative data system that included study team notes, which was merged into Data Table 2 after study completion. Given that dates, but not time, were used for merging, there was high confidence in this merging approach. Subsequent data checks provided quality assurance of this approach.

6.3.4 Merging use with encounters

There was no existing unique key to match MyChart Bedside use to hospital encounter data. To merge inpatient encounters with MyChart Bedside usage data, the merged encounter data discussed in 6.3.2 was used to establish admission and discharge dates and times. In these cases, the timestamp on the MyChart Bedside audit files was matched and used to link a user action to a specific encounter. Some user actions, approximately 1.5% of the raw MyChart Bedside log files, fell outside the encounter periods defined using the ADT record. Any actions that occurred outside an encounter period were dropped from further analyses.

6.3.5 MyChart Bedside user actions

MyChart Bedside log file data included 58 total actions that a user could perform. These actions could be classified into two categories: active and passive actions. Active actions were user initiated, required user engagement, and had exclusive and reliable representation in the log file across the entire study time frame. For example, sending a message action only occurred when an individual initiated a message send in MyChart Bedside. Passive actions occurred automatically, were non-exclusive, and were unreliable. For example, every five minutes the tablet refreshed telemetry data through no action of the patient. As a result, the research team sought to identify all active actions and associated them with one of the 10 functions offered in MyChart Bedside.

6.3.6 Survey data cleaning

Occasionally, respondents to the paper survey chose multiple responses to single response question. Such responder error was treated as non-response.

6.3.7 Merging billing data with encounters

Patient encounters and billing data may not have merged uniquely, as a single hospital charge record often incorporated multiple hospital encounters. Approximately 4.6% of the encounter dataset had billing data associated with multiple encounters. Additionally, because of the processing choice to merge close and overlapping encounters, multiple charges could have been associated with a single encounter. While the billing data was neither reported nor associated with a primary or secondary outcome, the study team thought it important to disclose its existence as part of this protocol document, as subsequent exploratory data analysis in other papers may refer to this data.

6.3.8 Additional exclusions

Several cases were identified post-hoc that created additional exclusions:

1. A negligible number of patients were enrolled in the study and in a subsequent visit were admitted as prisoners. In these cases, the enrolled participants were dropped from the analysis.

2. Study participants were dropped from the data if they withdrew from the study and were enrolled for less than three days. The study team determined that in these cases, the patient effectively indicated the intent to not be part of the study, despite originally agreeing to participate in the research.
3. Study participants with a length of stay less than three days were also dropped from the analysis. In prior work, it was determined that provisioning of tablets was not consistent for patients with length of stay less than three days.[20]
4. Study participants with no MyChart Bedside use in their enrollment admission were excluded from analysis. These cases may have occurred because an individual was enrolled into the study using a paper survey, but then never received their tablet.

6.3.9 Multiple surveys

Initial survey data processing revealed that some patients began multiple Admission surveys. Specifically, 322 patients had more than one survey, with a total of 719 multiple surveys. These cases were commonly related to technical issues with the Qualtrics survey system experienced by the patient, for example in the case of a loss of wireless connectivity. In these cases, patients would frequently log back into the system and attempt to complete the survey again. The research team developed a set of heuristics to determine which survey responses to keep (see Table 3).

Table 3. Multiple survey heuristics.

Rule	Cause	Description	Potential reason	Number of surveys dropped
**1**	Empty surveys	If a patient had multiple surveys and at least one was non-empty, the empty surveys were dropped.	Loss of connectivity could have created multiple empty survey records.	228
**2**	Paper surveys	When a patient had both electronic and paper survey, the electronic survey was dropped.	Patients started an electronic survey and then changed their preference and asked for paper.	94
**3**	Intervention check	When a patient had more than one survey attempt, the survey attempt that occurred in the same admission as the study intervention or interaction with the study team was kept.	Patients were discharged before the study team was able to perform an intervention. Subsequently, they were readmitted, attempted a new survey, and received an intervention or interacted with the study team. The first survey was discarded.	11
**4**	Recent admission	When a patient had more than one survey, the survey attempt from the most recent admission was kept.	Patients with multiple admissions and multiple survey attempts that did not receive an intervention or interaction with the study.	4
**5**	Completion rate	For patients who still had more than one survey attempt, only surveys with greater than 60% completion rate were kept.	Patients with multiple survey attempts within a single admission.	5

In the 71 cases where a participant completed more than one survey at greater than 60%, the research team adopted the following question level heuristic to identify the patient’s answer:

1. If a question was completed in only one survey attempt, that response was kept
2. If a question was the same in all survey attempts, that response was kept
3. If a question response was ever in conflict, that response was treated as missing

6.3.10 Missing Touch intervention date

The date of receipt of a High-Touch intervention was supposed to be recorded for each patient in Qualtrics in an administrative data system. In rare instances, this date was missing. For these instances, intervention delivery date was identified from study notes made by the TNs about patient visits that included dates and details of encounters and was entered manually.

Section 7: References

1. Bodenheimer T, Lorig K, Holman H, Grumbach K. Patient self-management of chronic disease in primary care. JAMA 2002;288:2469-75.
2. Coleman MT, Newton KS. Supporting self-management in patients with chronic illness. Am Fam Physician 2005;72:1503-10.
3. Smith SM, Soubhi H, Fortin M, Hudon C, O'Dowd T. Managing patients with multimorbidity: systematic review of interventions in primary care and community settings. BMJ 2012;345:e5205.
4. US Department of Health Human Services. Multiple chronic conditions–a strategic framework: optimum health and quality of life for individuals with multiple chronic conditions. Washington, DC: US Department of Health and Human Services 2010;2.
5. Wu S, Green A. Projection of chronic illness prevalence and cost inflation. Santa Monica, CA: RAND Health; 2000.
6. Demiris G, Afrin LB, Speedie S, et al. Patient-centered applications: use of information technology to promote disease management and wellness. A white paper by the AMIA knowledge in motion working group. J Am Med Inform Assoc 2008;15:8-13.
7. Halamka JD, Mandl KD, Tang PC. Early experiences with personal health records. J Am Med Inform Assoc 2008;15:1-7.
8. Hess R, Bryce CL, Paone S, et al. Exploring challenges and potentials of personal health records in diabetes self-management: implementation and initial assessment. Telemed J E Health 2007;13:509-17.
9. Kaelber DC, Jha AK, Johnston D, Middleton B, Bates DW. A research agenda for personal health records (PHRs). J Am Med Inform Assoc 2008;15:729-36.
10. Wald JS, Middleton B, Bloom A, et al. A patient-controlled journal for an electronic medical record: issues and challenges. Stud Health Technol Inform 2004;107:1166-70.
11. Agarwal R, Anderson C, Zarate J, Ward C. If we offer it, will they accept? Factors affecting patient use intentions of personal health records and secure messaging. J Med Internet Res 2013;15:e43.

12. Holtrop JS, Corser W, Jones G, Brooks G, Holmes-Rovner M, Stommel M. Health behavior goals of cardiac patients after hospitalization. Am J Health Behav 2006;30:387-99.
13. Lau K, Freyer-Adam J, Gaertner B, Rumpf HJ, John U, Hapke U. Motivation to change risky drinking and motivation to seek help for alcohol risk drinking among general hospital inpatients with problem drinking and alcohol-related diseases. Gen Hosp Psychiatry 2010;32:86-93.
14. Sieck CJ, Heirich M, Major C. Alcohol counseling as part of general wellness counseling. Public Health Nurs 2004;21:137-43.
15. Chodosh J, Morton SC, Mojica W, et al. Meta-analysis: chronic disease self-management programs for older adults. Ann Intern Med 2005;143:427-38.
16. Warsi A, Wang PS, LaValley MP, Avorn J, Solomon DH. Self-management education programs in chronic disease: a systematic review and methodological critique of the literature. Arch Intern Med 2004;164:1641-9.
17. Adler-Milstein J, Sarma N, Woskie LR, Jha AK. A comparison of how four countries use health IT to support care for people with chronic conditions. Health Aff 2014;33:1559-66.
18. Archer N, Fevrier-Thomas U, Lokker C, McKibbon KA, Straus SE. Personal health records: a scoping review. J Am Med Inform Assoc 2011;18:515-22.
19. Or CK, Tao D. Does the use of consumer health information technology improve outcomes in the patient self-management of diabetes? A meta-analysis and narrative review of randomized controlled trials. Int J Med Inform 2014;83:320-9.
20. McAlearney AS, Sieck CJ, Hefner JL, et al. High touch and high tech (HT2) proposal: transforming patient engagement throughout the continuum of care by engaging patients with portal technology at the bedside. JMIR Res Protoc 2016;5:e221.

High Tech and High Touch (HT2): Transforming patient engagement throughout the continuum of care by engaging patients with portal technology at the bedside

Statistical Analysis Plan

Plan Prepared By:
Daniel M. Walker, PhD, MPH[1,2]; Gennaro Di Tosto,[2]; Timothy R. Huerta, PhD, MS[1,2,3,4]; Ann Scheck McAlearney, ScD, MS[1,2,3,4]

[1]Department of Family and Community Medicine, College of Medicine, The Ohio State University; [2]CATALYST, Center for the Advancement of Team Science, Analytics, and Systems Thinking, College of Medicine, The Ohio State University; [3]Department of Biomedical Informatics, College of Medicine, The Ohio State University; [4]Division of Health Services Management and Policy, College of Public Health, The Ohio State University

Version 3.2 from November 10, 2023.

Table of Contents

Section 1: Statistical analysis 3

1.1 Confidence intervals and p-values 3

1.2 Missing data 3

1.3 Statistical software 3

1.4 Assumptions and testing 3

1.5 Hypothesis 1: Inpatient portal use 5

Section 2: Changes to the Statistical Analysis Plan 8

Section 3: References 9

Section 1: Statistical analysis

1.1 Confidence intervals and p-values

For all statistical tests, a p-value of 0.05 was used to assess significance and 95% confidence intervals were reported. No adjustment was done for multiple hypothesis testing.

1.2 Missing data

All available data was used for the analysis. Missing data was treated as missing completely at random (MCAR). No imputation was used.

1.3 Statistical software

All analyses were conducted using Stata MP 14.2.

1.4 Assumptions and testing

1.4.1 Analysis populations

Estimates of the intervention effects were calculated on two analytic populations. The main analytic population of the study used an *intention-to-treat (ITT)* approach. Using this paradigm, participants were analyzed on the basis of the study group to which they were randomized, regardless of their eventual receipt of that intervention.

The effects of the intervention were additionally evaluated on a *per-protocol* population, composed exclusively by those participants who were administered their randomized intervention. The analysis of the data from the per-protocol population was weighted by the inverse of their probability to receive the intervention to which they were originally randomized, computed on the basis of the following demographic and clinical characteristics: sex, race, age at time of enrollment, Charlson Comorbidity Index, length of stay.

1.4.2 Analytic framework

A review of factorial randomized control trials identified that the appropriate analytics model, in general, would adhere to Equation 1 (see below), assuming that the basic functional form for regression is the appropriate approach. This approach was used per Montgomery et al.[1] Variations to this functional form are noted below.

Eq. 1: $y = X_{Tech} + X_{Touch} + X_{Race} + X_{Tech*Touch} + X_{Tech*Race} + X_{Touch*Race} + X_{Tech*Touch*Race}$

To address potential differences in variance associated with the multi-site and pragmatic aspects of the study (e.g., expansion of the study after it was originally conceptualized and operationalized, differences in policies and procedures across hospitals and units), we performed cluster-robust standard errors in the estimation of the outcomes.[2] The analysis clusters study participants by the hospital where the study participant was enrolled. To validate this assumption, a sensitivity analysis was performed on patients transferred to different hospitals within the medical center.

Further, to account for heterogeneity in duration of patient access to the inpatient portal, we included the length of tablet provisioning, measured in days, as an offset parameter in all models.

1.4.3 Sub-sample analysis

We performed sub-sample analysis on the study participants who received the Full-Tech intervention (see Equation 2).

Eq. 2: $$If\ X_{Tech} = Full;\ y = X_{Touch} + X_{Race} + X_{Touch*Race}$$

1.4.3 Distributional assumptions

Prior to analysis, we will conduct an examination of the data to assess model fit for each hypothesis (i.e., Poisson, Negative Binomial, etc.).[i]

---

[i] Preliminary analysis revealed wide variance in the length of time that patients had a tablet, which we termed ‘provision length’. This provision length was included as an exposure in the model fitting process. Analysis after data collection suggested that the ANOVA model was a poor choice for the distribution of the data. Poisson models were excluded because of the foundational assumption–namely that the mean and variance of the response variable are the same. In the data, the variance was significantly greater than the mean, leading this model to significantly underestimate true variability in the data. Subsequent outlier analysis suggested no improvement. The negative binomial model was specified four ways: (1) negative binomial; (2) negative binomial with the provision length adjustment; (3) zero truncated; (4) zero truncated with the provision length adjustment. The negative binomial models appeared to provide a better fit relative to the other model choices. The zero truncated model with length of provision as an offset variable provided the best relative fit when comparing information criteria values. However, this improvement was viewed as marginal relative to the negative binomial model specification. For this reason and to account for the heterogeneity in provision length, all count outcomes were fit using a negative binomial model with the provision length adjustment.

1.4.5 Defining MyChart Bedside use sessions

A new MyChart Bedside use session was identified in the log files in two ways: (1) a "identify user with lock" action; or (2) any user action that occurred more than 15 minutes after the previous action. Some session periods contained no active tasks as defined in Section 6.3.5 in the Final Protocol. These inactive sessions were dropped from analyses. The "identify user with lock" action that marked the beginning of a session sometimes occurred multiple times sequentially without any other actions occurring. Retaining these recurring actions would create sessions with only a login action. To eliminate this problem, sequential "identify user with lock" actions were dropped.

1.4.6 Defining MyChart use sessions

MyChart log files contained timestamped records of patient actions on the outpatient portal. This data was processed to obtain information about the number of sessions associated with each unique study participant. For MyChart, a session was defined by a sequence of actions linked to a patient's medical record number (MRN), with the first action typically being a 'login' and the last one a 'logout'. In the case of MyChart sessions, at times patients did not actively logout of the application. Using the protocol described by Huerta, et al.,[3] we imposed a limit of 22 minutes as the length of time a patient was allowed to stay inactive before a 'logout' was imputed into the data. All actions occurring after the time limit were considered part of a new session. Additionally, sessions of length zero were discarded from the dataset.

1.4.7 Facility Transfer Sensitivity Analysis

Baseline characteristics were summarized, including age (median), gender (male, female percentages), race (White, Black, Other percentages), length of stay (days), and the Charlson comorbidity index (median, treated as a continuous score). Baseline characteristics were compared using analysis of variance (ANOVA) or t-tests, as appropriate, to examine equivalence between the four study arms and the impact on the model estimates for patients who were transferred between facilities was negligible.

1.5 Hypothesis 1: Inpatient portal use

H1: The difference in use of the MyChart Bedside inpatient portal between racial subgroups will be less among those assigned to the High intervention levels for Tech and Touch.

We operationalized higher use in this hypothesis in two ways:

1. Frequency of use
2. Comprehensive use

1.5.1 Outcome 1 – Number of MyChart Bedside sessions within the enrollment admission was dependent on treatment

Dependent variable specification: The count of MyChart Bedside sessions for the admission associated with study enrollment.

- Enrollment admission was defined as the admission when the patient was enrolled in the study.

Statistical model: Negative binomial model with non-interacted and interacted study arms as binomial predictors.

Covariates: No covariates were added to this analysis.

1.5.2 Outcome 2 – Comprehensive use was dependent on treatment

Dependent variable specification: A binary variable identifying participants that used all available functions based on their Tech arm assignment (1 = comprehensive functions user; 0 = not a comprehensive functions user).

- The definition of “comprehensiveness” was tested in two different ways:
  - **Definition 1: Based on use of all available functions**: Users were considered comprehensive functions users when they reached a threshold number of functions used based on their Tech Arm. For Lite-Tech users, this threshold was three selectable functions available–Bedside Tutorial, Dining on Demand, and To Learn. For Full-Tech users, this threshold was eight of the 10 functions available, including the three functions available to Lite-Tech users.
  - **Definition 2: Based on use of the functions available to all users**: Users were considered comprehensive functions users when they used the three functions that were available to all users.

Statistical model: Logistic regression model with non-interacted and interacted study arms as binomial predictors.

Covariates: No covariates were added to this analysis.

1.5.3 Outcome 3 – MyChart Bedside use (by function) was dependent on Touch arm.

Sub-sample specification: This analysis only used the Full-Tech arms, and therefore only used the Touch predictor in the statistical model (see Section 1.4.2).

Outcome specification: Proportion of total use, calculated for each user by dividing the sum of actions in a given function from the total sum of user actions during their enrollment admission accounted for by each function.

Statistical model: For each function, a fractional logistic regression model was performed, with use proportion as the response variable and Touch status as the explanatory variable.

Covariates: No covariates were added to this analysis.

Section 2: Changes to the Statistical Analysis Plan

The original statistical analysis plan was specified based on assumptions about the data collection and distributions of the data, and was designed to be exploratory and flexible to account for violation of modeling assumptions. The final statistical analysis plan reflects a more detailed description of how the analyses were performed.

Section 3: References

1. Montgomery AA, Peters TJ, Little P. Design, analysis and presentation of factorial randomised controlled trials. *BMC Med Res Methodol* 2003;3(1):26. doi: 10.1186/1471-2288-3-26
2. Barrios T, Diamond R, Imbens GW, et al. Clustering, Spatial Correlations, and Randomization Inference. *Journal of the American Statistical Association* 2012;107(498):578-91. doi: 10.1080/01621459.2012.682524
3. Huerta T, Fareed N, Hefner JL, et al. Patient engagement as measured by inpatient portal use: methodology for log file analysis. J Med Internet Res 2019;21(3):e10957. doi:10.2196/10957.

eAppendix 2. Original Trial Protocol

High Tech and High Touch (HT2): Transforming patient engagement throughout the continuum of care by engaging patients with portal technology at the bedside: Final Study Protocol

Plan Prepared By:
Ann Scheck McAlearney, ScD, MS[1,2,3,4]; Daniel M. Walker, PhD, MPH[1,2]; Naleef Fareed, PhD, MBA[2,3]; Lindsey Sova, MPH[2]; Cynthia J. Sieck, PhD, MPH[1,2]: Timothy R. Huerta, PhD, MS[1,2,3,4]

[1]Department of Family and Community Medicine, College of Medicine, The Ohio State University; [2]CATALYST, Center for the Advancement of Team Science, Analytics, and Systems Thinking in Health Services and Implementation Science Research, College of Medicine, The Ohio State University; [3]Department of Biomedical Informatics, College of Medicine, The Ohio State University; [4]Division of Health Services Management and Policy, College of Public Health, The Ohio State University

Version 2.0 (December 12, 2019):	Final pre-analysis Protocol
Version 2.1 (March 16, 2021):	Updated to reflect request from external reviewer to include sensitivity analysis.

Table of Contents

Section 1: Administrative information 4
- 1.1. Clinical trials registration 4
- 1.2 Ethics approval 4
- 1.3 Availability of data 4
- 1.4 Competing interests 4
- 1.5 Funding 4
- 1.6 Roles and responsibilities 4
- 1.7 Acknowledgements 4
- 1.8 Protocol revisions 5

Section 2: Introduction 9
- 2.1 Background and rationale 9
- 2.2 Objectives 9

Section 3: Study methods 11
- 3.1 Trial design 11
- 3.2 Randomization 14
- 3.3 Blinding 14
- 3.4 Sample size 15
- 3.5 Statistical interim analysis and stopping guidance 15
- 3.6 Timing of final analysis 15
- 3.7 Study flow 15
- 3.8 Timing and mode of outcome assessment 15

Section 4: Statistical principles 18
- 4.1 Confidence intervals and p-values 18
- 4.2 Adherence and protocol deviations 18
- 4.3 Analysis populations 19

Section 5: Trial population 20
- 5.1 Screening and eligibility 20
- 5.2 Recruitment 21
- 5.3 Study withdrawal 22
- 5.4 Baseline patient characteristics 22

Section 6: Data management 23

6.1 Data sources 23
6.2 Data model 23
6.3 Data processing 23
Section 7: References 28

Section 1: Administrative information

1.1. Clinical trials registration

This study is registered with clinicaltrials.gov: NCT02943109.

1.2 Ethics approval

The Institutional Review Board of The Ohio State University (OSU), College of Medicine (COM) approved the study.

1.3 Availability of data

The data used in this study includes identifiable information, and per our consent approval we are not permitted to share study data. However, the data management protocol and analytic approach will be made publicly available via publication.

1.4 Competing interests

The authors declare that they have no competing interests to report.

1.5 Funding

This work was supported by the Agency for Healthcare Research and Quality (AHRQ) Grants R01HS024091, R21HS024767, P30HS024379. While this research was funded by AHRQ, the study sponsor had no involvement in the collection, analysis, or interpretation of data, in the writing of this article, or in the decision to submit the article for publication.

1.6 Roles and responsibilities

ASM, TH, JH, CS, DW, AA, LS, MR, SMB designed the study, and with NF, GD, SRM, and AG developed and approved the initially submitted version of this manuscript. DW and LS wrote the Protocol, and TH was the senior statistician responsible for the Protocol. ASM was the Principal Investigator of the study. All authors read and approved the final manuscript.

1.7 Acknowledgements

The authors would like to thank the following individuals who were affiliated with the authors' organization during the project for their assistance:

Technical Support: Robert Taylor, Lakshmi Gupta, Seth Scarborough

Research Associates: Ayanna Scott, Jaclyn Volney, Toby Weinert, Alison Silverman, Danijela Cvijetinovic, Karen Alexander, Kimberly Karels

Administrative: Pamela Thompson

1.8 Protocol revisions

Revision history of the protocol, including rationale, is provided in Table 1, sorted by date.

Table 1: Amendments to the study protocol (sorted by date).

Date / Section reference	Revision	Rationale
**February 2016**<br>**Section 3.1**	**Low-Tech intervention:**<br>Changed from no tablet to Lite-Tech intervention with a tablet with a limited MyChart Bedside feature set.	Independent of the study, OSUWMC decided to implement MyChart Bedside system-wide, resulting in it not being possible to not provide some patients a tablet.
**February 2016**<br>**Section 3.4**	**Study site:**<br>Changed study sites from six matched unit pairs to OSUWMC system-wide, including six hospitals.	Independent of the study, OSUWMC decided to implement MyChart Bedside system-wide, resulting in it being feasible to expand the study sites across the OSUWMC.
**February 2016**<br>**Section 5.1**	**Focus on multiple chronic conditions:**<br>Changed from inclusion criteria of two or more chronic conditions to no disease restrictions.	Independent of the study, OSUWMC decided to implement MyChart Bedside system-wide, resulting in it being infeasible to restrict the sample based on disease characteristics.
**February 2016**<br>**Section 5.1**	**Inclusion criteria:**<br>Criteria of patient admitted within last 24 hours and expected discharge in more than 72 hours removed.	The expansion of the study from the six matched unit pairs to OSUWMC system-wide resulted in logistic challenges in being able to reaching all patients quickly. Additionally, OSUWMC does not store expected discharge in its Epic Clarity tables, making this criterion impossible to operationalize.
**February 2016**	**Low-Touch intervention:** Changed from active control where patients receive an in-person visit from a Technology Navigator (TN) to a passive control where patients were directed to self-guided learning materials built into MyChart Bedside.	Independent of the study, OSUWMC decided to implement MyChart Bedside system-wide. This change increased the number of potentially eligible patients for TNs to visit, presenting a logistical challenge. Additionally, in the original protocol where patients assigned to the Low-

**Section 3.1**		Tech arm did not receive a tablet, it was not possible to deliver a passive control. However, when the protocol changed to all study participants receiving a Tablet (Full/Lite-Tech), a passive control arm was possible. This approach resolved the logistical challenges.
**December 2016**<br><br>**Section 2.2**	**Hypotheses:**<br>The primary and secondary hypotheses were revised and reduced from seven to four hypotheses.	In the original proposed study, the plan was to execute it using 12 units and limit the study to chronic diseases. Additionally, participants were to be randomized to receive no technology or a full version of the MyChart Bedside application on the tablet. OSUWMC made several decisions that had an effect on the study design. In particular, OSUWMC determined that not providing a tablet was no longer an option; however, they made the units across the entire hospital system available for the study. The research team worked with OSUWMC to refine the hypotheses needed to reflect the more inclusive study. This resulted in dropping readmissions and comfort with technology as outcomes of interest, among other revisions.
**July 2017**<br><br>**Section 4.2.1**	**Improve enrollment yield:**<br>A research team member may visit the patient to remind them to complete the survey, limited to three visits per patient, once per day. Patients without completed surveys could also be visited on subsequent hospital admissions to complete their survey.	Quality assurance testing of the data collection system identified a technical problem which caused an unusual pattern of missing responses. Inspection of completeness of questions identified a pattern of non-completion such that every 5-10 questions, a question would demonstrate a considerably lower percent missing than the questions asked immediately before and after it. This pattern was attributed to the display of the survey on Qualtrics that hid some questions from view. A reminder was added to the survey to inform respondents if they had left questions unanswered as they clicked the button to advance

		the survey, still allowing them to advance if that was their preference.
**July 2017**<br><br>**Section 4.2.1**	**Study enrollment:**<br>A confirmation was added to each page of the survey to inform respondents when they had left questions unanswered on a page. They could continue to advance if that was their preference.	Completion of the survey was found to limit the number of patients in the High-Touch study arm. This change represented a deviation from the original protocol which had required that the Admission survey be completed prior to intervention delivery.
**February 2018**<br><br>**Section 3.1.3**	**Study allocation:**<br>The allocation of individuals in arms were rebalanced to ensure sufficient numbers of participants in the High-Touch treatment arm due to a significant number of patients becoming lost to follow-up.	Quality monitoring of the study found a number of exogenous factors that were impacting the High-Touch intervention, including, but not limited to: patient discharges; patient moves to other rooms; patients not present during rounding; patients' engagement with the care team; and tablet returns prior to receiving an intervention. This change was done to ensure that sufficient individuals were assigned to the High-Touch intervention to increase the number of patients who would be eligible to receive a High-Touch intervention.
**March 2018**<br><br>**Section 4.2.1**	**Intervention available after consent:**<br>TNs are allowed to make first contact for the High Touch intervention once the patient consented – no longer waiting for a complete Admission Survey. The TN should work with the patient to walk them through completing the survey during the intervention.	As noted in Section 3.1.3 changes were made to the intervention protocol to facilitate greater engagement in the High-Touch arm. Specifically, prior to February 2018, participants assigned to the High-Touch intervention group did not receive the intervention until after they had completed the Admission survey. Starting on February 22, 2018, the research team modified the protocol to allow TNs to mark a survey as complete and allow the intervention team to perform the High-Touch intervention. In March 2018, 22 days later, the protocol was once again changed to deliver the High-Touch intervention at first contact with the TN if they had been assigned to the High-Touch intervention.

**July 2018**<br><br>**Section 4.2.1**	**Intervention assignment:** Individuals that had activated a MyChart Bedside account prior to the study start or in a Full-Tech unit were manually assigned to the Full-Tech intervention regardless of their MRN. Individuals who were found to have accessed both Full and Lite versions over the course of the study were excluded from the analysis for this study.	Quality assurance testing of ongoing data collection of the MyChart Bedside usage data revealed that patients who were to have been assigned to Lite-Tech based on their MRN were, in fact, using functions only available on Full-Tech. Subsequent review also found the reverse to be true, participants that were assigned to Full-Tech were limiting their usage to those functions in Lite-Tech.<br><br>The study team conferred with Epic– the electronic health record (EHR) vendor that developed the code to assign patients to a particular arm of Tech status–and manually reconstructed Tech assignment using the original assignment code. A cohort of individuals were identified as not having received the intended Tech assignment. Through this process, the study team was able to secure the true Tech intervention status. Individuals that had activated a MyChart Bedside account or were in a Full-Tech unit prior to the study start were manually assigned to Full-Tech regardless of their MRN. Additionally, individuals who were found to have accessed both Full and Lite versions over the course of the study were identified and subsequently excluded from the analysis for this study. This scenario may have occurred when a patient was assigned an MRN at admission but subsequent records review identified an existing MRN. In these cases, individuals unexpectedly transitioned between two Tech intervention states.

Section 2: Introduction

2.1 Background and rationale

For patients with complex care needs, engagement in disease management activities is particularly critical.[1-4] The human cost of chronic illnesses touches almost every person in the US. These costs are real, personal and pervasive.[5] In response, patients often seek out tools to help them manage their health. Patient portals–a Personal Health Record (PHR) tethered to an Electronic Health Record (EHR)–show promise as tools that patients accept and value and that can improve health.[6-10] Although patient portals currently focus on the outpatient experience, the Ohio State University Wexner Medical Center (OSUWMC) has deployed a portal designed specifically for the inpatient experience that is connected to the ambulatory patient portal available after discharge. While this inpatient technology is in active use at only one other hospital in the US, healthcare facilities are currently investing in the infrastructure necessary to support large-scale deployment.[11]

Times of acute crisis such as hospitalization may increase a patient's focus on his/her health, a factor that has been linked in other areas to a greater awareness of health risks and increased focus on health behaviors.[12-14] During this time, patients may be more engaged with their care and therefore particularly interested in the potential for using tools to manage their health after discharge. Evidence shows that enhanced patient self-management can lead to better control of chronic illness,[1,15,16] and patient portals may serve as a mechanism to facilitate increased engagement.[17-19]

Our proposed four-year study used a mixed-methods approach to evaluate a pragmatic randomized controlled trial (RCT) studying the effectiveness of a High-Tech Intervention (MyChart Bedside, the inpatient portal), and an accompanying High-Touch Intervention (training patients to use the portal to manage their care and conditions) in a sample of hospitalized patients. This study measured how a patient portal tailored to the inpatient stay could improve patient experience and increase patient engagement by 1) improving patients' perceptions of the process of care while in the hospital, 2) increasing patients' self-efficacy for managing their health and health care, and 3) facilitating continued use of a patient portal for care management activities after discharge. In addition, we aimed to enhance patients' use of the patient portal available to outpatients, MyChart, once they were discharged.

2.2 Objectives

The ***High Tech and High Touch (HT2): Transforming patient engagement throughout the continuum of care by engaging patients with portal technology at the bedside*** study at OSU Wexner Medical Center (OSUWMC) sought to study how the deployment of a commercial (i.e., Epic Systems) inpatient portal, MyChart Bedside, impacted identified patient outcomes. To evaluate the impact of function set and training

on inpatient (i.e., MyChart Bedside) and outpatient (i.e., MyChart) portal use, the HT2 Study[20] was designed as a large-scale, pragmatic randomized controlled trial across six hospitals in a healthcare system.

Using a two-by-two factorial design, HT2 simultaneously examined the effects of two approaches to portal training with two versions of the inpatient portal. HT2 was designed to test four study hypotheses:

Hypotheses related to primary outcomes:

> *H1: Patients assigned to the High intervention levels for Tech and Touch will demonstrate higher use of MyChart Bedside.*
>
> *H2: Patients assigned to the High intervention levels for Tech and Touch will demonstrate higher levels of satisfaction with (H2a) and involvement in (H2b) their care experience.*

Hypotheses related to secondary outcomes:

> *H3: Patients assigned to the High intervention levels for Tech and Touch will demonstrate higher rates of outpatient portal adoption (i.e., of Epic's MyChart) for those admitted without prior outpatient portal use (H3a), and higher use of MyChart for those admitted who had previously used the outpatient portal (H3b).*
>
> *H4: Patients assigned to the High intervention levels for Tech and Touch will demonstrate higher levels of self-efficacy.*

To prevent outcome reporting bias and data-driven analysis of results, we produced this detailed statistical analysis plan. The plan was originally developed and drafted prior to examination of the data, and in the absence of randomization knowledge. The plan has been revised as the analysis has proceeded.

Section 3: Study methods

3.1 Trial design

This study was designed as a pragmatic factorial randomized controlled trial of two concurrent patient portal-related interventions.

3.1.1 Intervention description: Tech - Full/Lite

The Full-Tech intervention consisted of the standard build of the MyChart Bedside product as delivered from Epic and augmented by OSUWMC at the start of the study. The application was served by a *Home* landing page that displays events scheduled for the day and recent health updates and included access to 10 additional functions located in tabs along the side of the inpatient portal image:

1. *Home*: presented events scheduled for the day and data on patient vitals, including last blood pressure reading, current pulse and other patient vital signs refreshed from the Electronic Health Record (EHR) once every 5 minutes[i]
2. *Bedside Tutorial*: tutorial materials for the use of MyChart Bedside, including an 11-minute video explaining how to use MyChart Bedside and access to PDF versions of tutorial materials
3. *Dining on Demand*: food ordering service
4. *To Learn*: educational resources for the patient based on their treatment plan
5. *Happening Soon*: a timeline for the care activities that the patient could expect for the day
6. *Taking Care of Me*: information detailing members of the patient's care team
7. *Messages*: non-urgent secure messaging with the patient's care team
8. *My Health*: recent vital signs and lab test results
9. *Notes*: write/record reminders and notes
10. *I Would Like*: non-urgent requests from pastoral care or gift shop
11. *OSU MyChart*: allowed the patient to access MyChart, including creating a new account

In addition to the *Home* landing page, the Lite-Tech version of MyChart Bedside consisted of the 3 functions: *Bedside Tutorial*, *Dining on Demand*, and *To Learn*.

3.1.2 Intervention description: Touch - High/Low

The education and training intervention, referred to as Touch, was performed by trained Technology Navigators (TNs) who deliver training tailored to both the version of

[i] This function was automatic, so was not considered selectable and not included in the count of functions for either the Full- or Lite-Tech versions.

MyChart Bedside installed on their tablet (both Full- and Lite-Tech) and the perceived capacity of the patient.

Patients assigned to the Low-Touch intervention received an intervention focused on providing them with resources to conduct self-guided learning of MyChart Bedside. Specifically, TNs directed patients to the tutorial videos integrated in the MyChart Bedside application that explained the portal's functions.

In contrast, patients assigned to the High-Touch intervention would systematically review each of the functions of MyChart Bedside with the TN who would discuss how and when they are appropriately used. Patients were encouraged to use their tablet and follow along with the training as the TN moved through each function and ask the TN questions about their records during the presentation. The TNs tailored their presentation based on a patient's demonstrated knowledge of the technology to minimize patient participation burden while focusing on explaining the value of each function within the application. Patients unavailable after three intervention attempts were converted to Low-Touch.

3.1.3 Allocation ratio

The Tech intervention contrasted two function sets–Lite-Tech (40% allocation), which was a minimalistic toolset focused on providing basic data to the patient, and Full-Tech (60% allocation), a more expansive toolset. The Touch intervention contrasted two training approaches–Low-Touch, a self-guided approach where patients could view built-in tutorials, and High-Touch (initially 50% allocation), where patients receive in-person guided training with the technology. The realized allocation ratios are presented in Table 2.

Table 2. Realized allocation ratio.

Tech Allocation	Touch Allocation		
	High	Low	Total
Full			
Frequency	624	1,523	2,147
Row %	29.1%	70.9%	100.0%
Column %	69.3%	76.5%	74.2%
Overall %	21.6%	52.7%	74.2%
Lite			
Frequency	277	468	745
Row %	37.2%	62.8%	100.0%
Column %	30.7%	23.5%	25.8%
Overall %	9.6%	16.2%	25.8%
Total			
Frequency	901	1,991	2,892
Row %	31.2%	68.8%	100.0%
Column %	100.0%	100.0%	100.0%
Overall %	31.1%	68.8%	100.0%

The assignment protocol used three phases where the Touch allocation ratio was changed by the research team.

1. In Phase 1, patients received the High-Touch intervention only after consenting to the study and submitting their Admission survey (see Section 3.8.2). Low-Touch patients could view the tutorial at any point and were not required to complete the Admission survey. Phase 1 assigned patients to High/Low-Touch in a 1:1 ratio.
2. In Phase 2, beginning in February 2018, the study shifted to a 3:1 ratio of High/Low-Touch. This change was done to ensure that sufficient individuals were assigned to the High-Touch intervention due to under-enrollment. Quality monitoring of the study found a number of exogenous factors that were impacting the High-Touch intervention, including, but not limited to: patient discharges; patient moved to other rooms; patient not present during TN rounding; patient was engaged with the care team and unable to work with the TN; and tablet returned prior to receiving an intervention. This change in the ratio was intended to increase the number of patients who would be eligible to receive a High-Touch intervention while maintaining the *per protocol* approach in that these exceptions were considered a component of the Protocol.
3. Phase 3 began in March 2018 and lasted through the end of the study in September, 2018. This phase maintained the 3:1 split, but removed the requirement that patients complete the Admission survey prior to receiving the High-Touch intervention. Completion of the survey was found to limit the number of patients in the High-Touch study arm. This change represented a deviation

from the original protocol which had required that the Admission survey be completed prior to intervention delivery (see Section 4.2.1).

3.2 Randomization

Participants were randomized into each arm of each intervention, Tech and Touch, in separate processes.

3.2.1 Tech randomization

Tech randomization was accomplished using a 4-digit random number table generated using Stata version 14.0 which was used as a comparison table against the last four digits of the patient's Medical Record Number (MRN) which was assigned prior to study assignment. A preliminary simulation analysis found that random assignment using this model created appropriately sized groups of 40% and 60%.

Individuals who had access to the Full version of MyChart Bedside prior to enrollment in the study (i.e., those patients who were admitted to the Cancer Center, which was not included as a site, or patients who had used MyChart Bedside during the pilot phase of implementation in which only Full-Tech was available) maintained their Full-Tech status and were excluded from randomization if they were transferred to a unit included in the study.

3.2.2 Touch randomization

The study protocol included a series of robust approaches to randomization. Generally, Touch randomization occurred at consent using the randomizer function in Qualtrics which was used for both consent and survey data collection. The Protocol included a procedure for the assignment of Touch in cases where the patient was checked in after daily census and was not included in the Qualtrics randomization (i.e., due to timing of reconciliation between report pulls and the start of the work day). In those cases, a predefined randomization table was used to assign Touch status.

3.3 Blinding

Both the standard and alternate processes for Touch assignment occurred prior to study consent and therefore the TN, the patient, and the care team were blind to their study assignment at consent. Additionally, at the time of provisioning, both the patient and the nurse that assigned the patient the tablet were blind to Tech assignment. TNs providing the intervention were blind to Tech assignment prior to consent, but not prior to study eligibility assessment.

3.4 Sample size

The study did not adopt a sampling frame and represented a census approach to the implementation. The Consort Diagram (see Figure 1), provides information on the pragmatic factors that limited enrollment.

The original protocol calculated power based on 2016 Hospital Consumer Assessment of Healthcare Provider and Systems (HCAHPS) patient satisfaction scores as a baseline for outcome measures using G*Power 3.1 software. An expected enrollment of 3,872 was planned with the design expected to be sensitive enough to identify changes of between 2.2% and 3.8% across the panel of HCAHPS patient satisfaction scores.

3.5 Statistical interim analysis and stopping guidance

No interim analyses were planned or conducted for our study. Since HT2 posed minimal risk, stopping guidance was not applicable.

3.6 Timing of final analysis

HT2 was a minimal risk study, and as a result, final outcomes and their association with the interventions were reviewed only upon study completion.

3.7 Study flow

Patient participation in the study followed the CONSORT diagram shown in Figure 1.

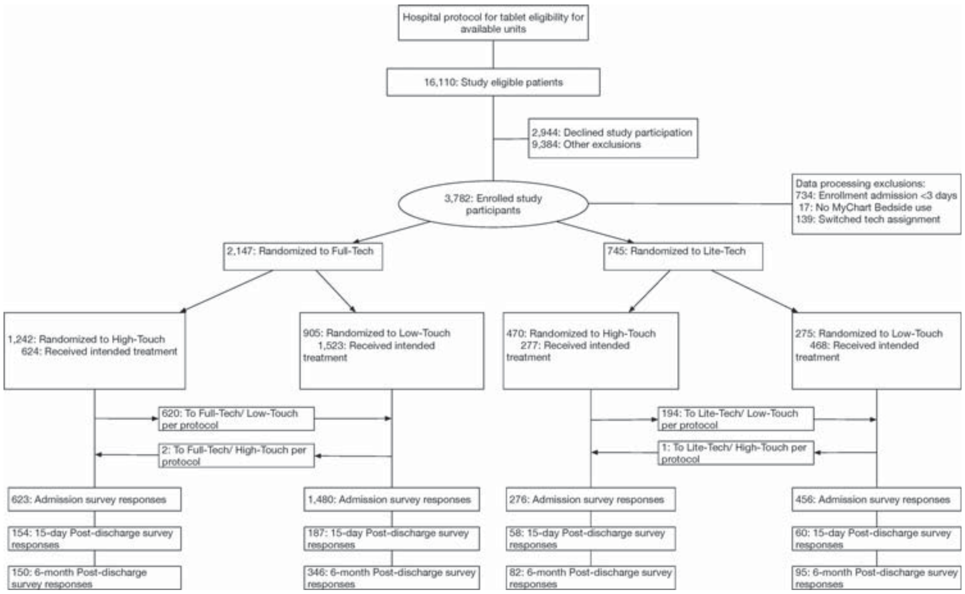

Figure 1. CONSORT diagram.

3.8 Timing and mode of outcome assessment

The outcomes for this study included measures of use of both the inpatient and outpatient portal as well as measures of satisfaction and self-efficacy.

3.8.1 Portal usage

MyChart Bedside and MyChart usage was determined through the use of log file analysis (LFA). The methods associated with LFA have been extensively discussed in methods papers published by the research team. Log files provided the date, time, and type of interaction with each of the functions in both of the patient portals. The log file data that was available included:

1. Login date and time
2. Functions accessed
3. Activity notes

Log files were linked to the patient MRN within the Epic Clarity clinical data record. MyChart Bedside and MyChart log files were obtained in an identified form for study participants for purposes of matching this data to Admission and Post-discharge surveys and follow-up interviews (see Section 6 - Data Management).

3.8.2 Satisfaction and self-efficacy

Data on satisfaction and self-efficacy were collected via survey at enrollment (referred to as the Admission survey), between 10 and 21 days post-discharge (referred to as the 15-day Post-discharge survey) and, finally, at six months post-discharge (referred to as the 6-month Post-discharge survey). The three surveys each gathered data on the outcomes to allow for comparisons at different time points.

The Admission survey included questions related to self-efficacy, hospital satisfaction, and patient experience. The Admission survey was administered in two modes: a paper form and electronically. The electronic survey was created and collected using Qualtrics online survey software. Patients accessed the survey via the MyChart Bedside portal. The default administration mode was electronic; paper forms were only given if the patient requested it or encountered a technical issue with the hospital-issued tablet that affected their ability to complete the survey electronically.

3.8.3 Post-discharge survey administration

Both the 15-day and 6-month Post-discharge surveys were administered based on the communication preference of the participant. During the consent process, participants were asked to identify a communication preference–email or phone–through which they would be contacted for the follow-up surveys. The study team developed a robust communication approach that would initiate contact based on stated preference, allow for participants to change their preference throughout the study, and would leverage alternate approaches if the participant could not be reached using their preferred approach. When email was used, the research team would send the participant an

invitation and a link to the Qualtrics online survey and up to three reminders. When a phone call was used, the research team would attempt up to three calls. Communication efforts for Post-discharge surveys began 10 days post-discharge from the enrollment admission and 24 weeks post-discharge from the enrollment admission.

Section 4: Statistical principles

4.1 Confidence intervals and p-values

For all statistical tests, a p-value of 0.05 was used to assess significance and 95% confidence intervals were reported. No adjustments were made for multiple hypothesis testing.

4.2 Adherence and protocol deviations

The HT2 study was designed as a pragmatic study with the interventions conducted using a *per protocol* approach.

Protocol deviations

Protocol deviations may have occurred if:

1. a participant consented to participate in the study but subsequently requested a change in Tech status to the OSUWMC;
2. a participant was admitted to the OSUWMC, was assigned to the Lite intervention, and was subsequently admitted to a unit that always received the Full version of MyChart Bedside.

In these cases, the individual was removed from the study.

4.2.1 Operational protocol amendments

Operational protocol amendment 1: Improve enrollment yield (Jan 2017)

A research team member could visit the patient to remind them to complete the survey, limited to three visits per patient, once per day. Patients without completed surveys could also be visited on subsequent hospital admissions to complete their survey.

Operational protocol amendment 2: Improve data quality (Jul 2017)

A confirmation was added to each page of the survey to inform respondents when they had left questions unanswered on a page. They could continue to advance if that was their preference.

Operational protocol amendment 3: Intervention available after consent (Feb 2018)

TNs were allowed to make first contact for the High-Touch intervention once the patient consented–no longer waiting for a complete Admission survey. The TN should work with the patient to walk them through completing the survey during the intervention.

Operational protocol amendment 4: Intervention assignment (Mar 2018)

Individuals that had activated a MyChart Bedside account or were in a Full-Tech unit prior to the study start were manually assigned Full-Tech regardless of their MRN.

Individuals who were found to have accessed both Full and Lite versions over the course of the study were to be excluded from the analysis for this study.

4.3 Analysis populations

The analytic population of the study used a *per protocol* approach. Using this paradigm, study participants assigned to the High-Touch group that did not receive the High-Touch intervention were reassigned to the Low-Touch group. In rare instances (n=3) where a patient was assigned to the Low-Touch group but received the training intervention due to patient request, they were reassigned to the High-Touch group.

Section 5: Trial population

5.1 Screening and eligibility

All admitted patients were screened at admission by nursing staff for eligibility to use a standardized tablet and the associated MyChart Bedside application based on the following protocol, developed by OSUWMC:

1. Patients must have been admitted to a hospital in the OSUWMC system where MyChart Bedside has been deployed.
2. Patients must have been capable of accepting the tablet terms of service.
3. Patients must not have had an excluded status, which were:
    a. less than 18 years old;
    b. legally blind;
    c. cannot speak and/or read English;
    d. involuntarily confined or detained; or,
    e. be considered as having a diminished decision-making capacity.

Nursing staff used their judgement in terms of whether a patient may have had an additional reason for exclusion and documented those reasons in the EHR. Acceptable reasons for exclusion included that the patient declined to use a tablet or the patient was not coherent at the time the nursing staff was rounding for the purpose of providing tablets. Patients were provided Android tablets by the medical center for use during their stay. The provisioning process defined the patient population and was outside of the scope of the study.

5.1.1 Inclusion criteria

Study inclusion criteria were as follows:

1. patient accepted tablet and use of MyChart Bedside;
2. patient was available in their room; and,
3. patient was capable of informed consent.

Only patients who were provisioned with MyChart Bedside were included in the study.

5.1.2 Exclusion criteria

Study exclusion criteria were as follows:

1. the patient was less than 18 years of age.

The inclusion criteria, by design, means that family and proxy users were excluded from the study.

5.2 Recruitment

The AMC Epic Clarity tables were used to develop a fully identified custom Census Report which provided information on all patients who met the inclusion criteria present in the hospital at midnight of each day. The research team integrated this report with the study database to create a daily list of individuals that had consented, refused, not been provided a tablet, or were eligible for being visited by a TN during TN rounding. This information was used to set up a rounding schedule to visit the rooms of these patients to follow up about study participation as follows:

1. In cases where a patient had not been provisioned and there were no clinical notes detailing a rationale for the withholding of access, members of the research team would approach the nursing team and ask them to make a formal determination about the appropriateness of the tablet for the patient.
2. In cases where a patient was provisioned, the study team would put the patient on the rounding schedule and attempt to recruit them to the study.

5.2.1 Study enrollment, participation, and remuneration

Eligible identified patients were invited to participate in the study via a link in MyChart Bedside which presented an electronic combined HIPAA authorization and consent form approved by the AMC's Institutional Review Board (IRB) for patients to sign to authorize extraction of identified clinical data. Study staff approached eligible patients to identify their interest in participation. Interested patients were guided to the tab within MyChart Bedside that explained that participation included the following tasks and consent:

1. a survey to be taken immediately upon completing consent (the Admission survey);
2. two surveys that would be taken by phone or via an emailed link;
    a. The 15-day Post-discharge survey
    b. The 6-month Post-discharge survey
3. additionally, patients were notified that they may be contacted for up to two telephone interviews: within 21 days of discharge and at approximately 6 months post-discharge. Not all individuals would be invited to participate in these interviews;
4. consent to share their clinical data, including the log files associated with their use of patient portals.

Upon completion of each survey, participants had the option to enter contact information on a separate page for a raffle of a $100 gift card and for future follow-up for additional surveys and interviews. One gift card was raffled off each week throughout data collection.

Participants were provided the option to request a paper combined HIPAA authorization and consent form to sign, delivered by a study team member. Participants received an electronic copy of the signed combined HIPAA authorization and consent form or a paper version from a study team member within 24 hours. If the patient had been discharged, a paper version was mailed to the address on file.

5.3 Study withdrawal

All data collected during the period as study participants was included in the analysis. Individuals who chose to withdraw were, in effect, refusing to contribute further data, including the following:

1. Patients who removed themselves from the study prior to discharge in the admission in which they are enrolled had no data about them included in the study.
2. Patients who removed themselves from the study after discharge in the admission in which they are enrolled had the Admission survey data and MyChart Bedside use data about them included in the study, but depending on when they withdrew, may not have any data about them included as part of either of the Post-discharge surveys.
3. Patients who were not reachable after discharge for the 15-day Post-discharge survey were not considered lost to follow-up, and contact was attempted for the 6-month Post-discharge survey.

5.4 Baseline patient characteristics

Baseline characteristics were summarized, including age (median), gender (male, female percentages), race (White, Black, Other percentages), length of stay (days), and the Charlson comorbidity index (median, treated as a continuous score). Baseline characteristics were compared using analysis of variance (ANOVA) or t-tests, as appropriate, to examine equivalence between the four study arms.

Section 6: Data management

6.1 Data sources

Data for this study was merged from two sources–one delivered from surveys promulgated by Qualtrics, a cloud-based survey system, and the other from the OSUWMC Epic Clarity tables. Data was kept in separate tables in accordance with the study data model.

6.2 Data model

Five tables were used in the data model used for this study. Figure 2 illustrates the entity data diagram for each table in the data model and their relationship.

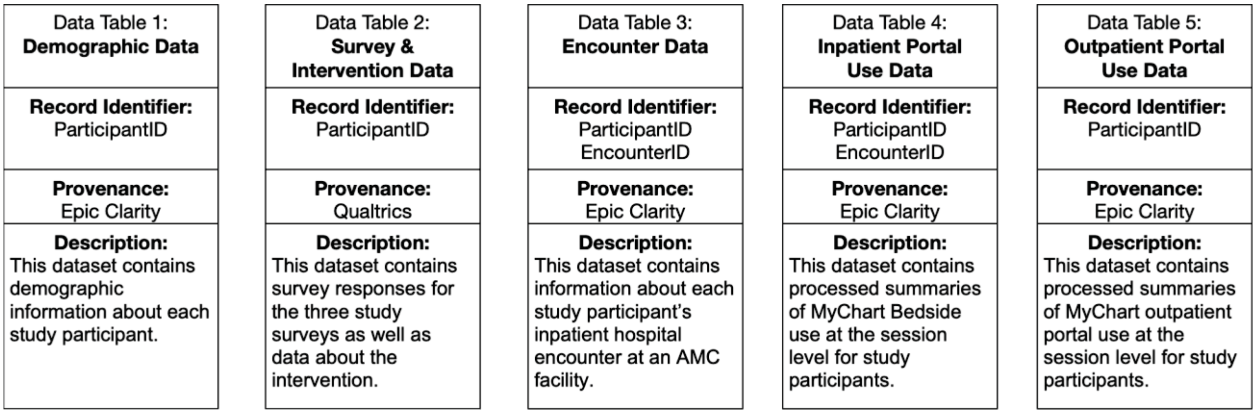

Figure 2. Entity data diagram.

Data Tables 1 and 2 provided data related to the participant. Intervention data was written into Qualtrics records from the EHR via a passthrough using a secure application programming interface (API). Data Tables 3 and 4 provided data about the patient's encounter history during the study period as well as their use of the inpatient portal for each visit. Data Table 5 provided data about the use of the outpatient portal, which therefore meant it was not related to a specific encounter. Where possible, the original data in the associated Epic Clarity tables were delivered to the research team, including the Admission/Discharge/Transfer (ADT) table. This approach was used to ensure that joins were completed by the research team as opposed to an intermediate data broker and ensure data quality.

6.3 Data processing

As noted in Section 6.2, data from the original Epic Clarity tables were provided to the research team. This process was deemed necessary, as quality testing of the data found a number of join errors that created issues when using the study and EHR derived data. We detailed ten specific data quality issues that were found and resolved in the data quality assurance testing.

6.3.1 Multiple MRNs

MRNs were used as primary identifiers by both the hospital and in the context of the study. Moreover, MRNs were used throughout data processing to associate data with a unique patient. Typically, each unique patient had a single, unique MRN. In rare cases, for instance when a patient presented in the emergency room and was unable to provide identification, a patient may have been temporarily issued an MRN. While hospital records are adjusted to ensure a single, unique MRN at a later time, data was written to the log files with the temporary MRN. Patients with multiple MRNs were identified, along with their duplicate MRNs, and the identifiers were recoded to a “primary” MRN in the processed data.

6.3.2 Combining hospital encounters

In preliminary data processing, it was discovered that the ADT report contained both overlapping admissions as well as two separate admissions within close time proximity. These scenarios commonly occurred when an individual moved facilities for diagnosis and treatment; for instance, in the case of an individual in the general hospital moving to the heart hospital for testing. To resolve these issues, data analysts merged admissions within 4 hours of each other, as well as overlapping admissions, and considered them a single admission. Data quality tests found that approximately 9% of discharge-subsequent admission and overlaps were resolved by this threshold. In cases where a discharge was recorded and a subsequent admission was recorded more than four hours later, that admission was deemed to be a new, separate admission.

6.3.3 Matching admission surveys to a patient encounter

Both the MRN and the date and time of the start of the Admission survey was used to match an Admission survey to a specific patient encounter in the ADT table. However, Admission surveys were collected using two modalities–electronic and paper–the latter of which did not produce an electronic timestamp for survey start. In the first case, electronic data capture via the Qualtrics-hosted survey, MRN, and survey start/stop times were written to Data Table 2, however in cases where paper surveys were used, no such recording of start/ stop time was automatically recorded. In this latter case, the date and estimated time the survey was delivered was recorded in a separate administrative data system that included study team notes, which was merged into Data Table 2 after study completion. Given that dates, but not time, were used for merging, there was high confidence in this merging approach. Subsequent data checks provided quality assurance of this approach.

6.3.4 Merging use with encounters

There was no existing unique key to match MyChart Bedside use to hospital encounter data. To merge inpatient encounters with MyChart Bedside usage data, the merged encounter data discussed in 6.3.2 was used to establish admission and discharge dates and times. In these cases, the timestamp on the MyChart Bedside audit files was matched and used to link a user action to a specific encounter. Some user actions, approximately 1.5% of the raw MyChart Bedside log files, fell outside the encounter periods defined using the ADT record. Any actions that occurred outside an encounter period were dropped from further analyses.

6.3.5 MyChart Bedside user actions

MyChart Bedside log file data included 58 total actions that a user could perform. These actions could be classified into two categories: active and passive actions. Active actions were user initiated, required user engagement, and had exclusive and reliable representation in the log file across the entire study time frame. For example, sending a message action only occurred when an individual initiated a message send in MyChart Bedside. Passive actions occurred automatically, were non-exclusive, and were unreliable. For example, every five minutes the tablet refreshed telemetry data through no action of the patient. As a result, the research team sought to identify all active actions and associated them with one of the 10 functions offered in MyChart Bedside.

6.3.6 Survey data cleaning

Occasionally, respondents to the paper survey chose multiple responses to single response question. Such responder error was treated as non-response.

6.3.7 Merging billing data with encounters

Patient encounters and billing data may not have merged uniquely, as a single hospital charge record often incorporated multiple hospital encounters. Approximately 4.6% of the encounter dataset had billing data associated with multiple encounters. Additionally, because of the processing choice to merge close and overlapping encounters, multiple charges could have been associated with a single encounter. While the billing data was neither reported nor associated with a primary or secondary outcome, the study team thought it important to disclose its existence as part of this protocol document, as subsequent exploratory data analysis in other papers may refer to this data.

6.3.8 Additional exclusions

Several cases were identified post-hoc that created additional exclusions:

1. A negligible number of patients were enrolled in the study and in a subsequent visit were admitted as prisoners. In these cases, the enrolled participants were dropped from the analysis.

2. Study participants were dropped from the data if they withdrew from the study and were enrolled for less than three days. The study team determined that in these cases, the patient effectively indicated the intent to not be part of the study, despite originally agreeing to participate in the research.
3. Study participants with a length of stay less than three days were also dropped from the analysis. In prior work, it was determined that provisioning of tablets was not consistent for patients with length of stay less than three days.[20]
4. Study participants with no MyChart Bedside use in their enrollment admission were excluded from analysis. These cases may have occurred because an individual was enrolled into the study using a paper survey, but then never received their tablet.

6.3.9 Multiple surveys

Initial survey data processing revealed that some patients began multiple Admission surveys. Specifically, 322 patients had more than one survey, with a total of 719 multiple surveys. These cases were commonly related to technical issues with the Qualtrics survey system experienced by the patient, for example in the case of a loss of wireless connectivity. In these cases, patients would frequently log back into the system and attempt to complete the survey again. The research team developed a set of heuristics to determine which survey responses to keep (see Table 3).

Table 3. Multiple survey heuristics.

Rule	Cause	Description	Potential reason	Number of surveys dropped
1	Empty surveys	If a patient had multiple surveys and at least one was non-empty, the empty surveys were dropped.	Loss of connectivity could have created multiple empty survey records.	228
2	Paper surveys	When a patient had both electronic and paper survey, the electronic survey was dropped.	Patients started an electronic survey and then changed their preference and asked for paper.	94
3	Intervention check	When a patient had more than one survey attempt, the survey attempt that occurred in the same admission as the study intervention or interaction with the study team was kept.	Patients were discharged before the study team was able to perform an intervention. Subsequently, they were readmitted, attempted a new survey, and received an intervention or interacted with the study team. The first survey was discarded.	11
4	Recent admission	When a patient had more than one survey, the survey attempt from the most recent admission was kept.	Patients with multiple admissions and multiple survey attempts that did not receive an intervention or interaction with the study.	4
5	Completion rate	For patients who still had more than one survey attempt, only surveys with greater than 60% completion rate were kept.	Patients with multiple survey attempts within a single admission.	5

In the 71 cases where a participant completed more than one survey at greater than 60%, the research team adopted the following question level heuristic to identify the patient’s answer:

1. If a question was completed in only one survey attempt, that response was kept
2. If a question was the same in all survey attempts, that response was kept
3. If a question response was ever in conflict, that response was treated as missing

6.3.10 Missing Touch intervention date

The date of receipt of a High-Touch intervention was supposed to be recorded for each patient in Qualtrics in an administrative data system. In rare instances, this date was missing. For these instances, intervention delivery date was identified from study notes made by the TNs about patient visits that included dates and details of encounters and was entered manually.

Section 7: References

1. Bodenheimer T, Lorig K, Holman H, Grumbach K. Patient self-management of chronic disease in primary care. JAMA 2002;288:2469-75.
2. Coleman MT, Newton KS. Supporting self-management in patients with chronic illness. Am Fam Physician 2005;72:1503-10.
3. Smith SM, Soubhi H, Fortin M, Hudon C, O'Dowd T. Managing patients with multimorbidity: systematic review of interventions in primary care and community settings. BMJ 2012;345:e5205.
4. US Department of Health Human Services. Multiple chronic conditions–a strategic framework: optimum health and quality of life for individuals with multiple chronic conditions. Washington, DC: US Department of Health and Human Services 2010;2.
5. Wu S, Green A. Projection of chronic illness prevalence and cost inflation. Santa Monica, CA: RAND Health; 2000.
6. Demiris G, Afrin LB, Speedie S, et al. Patient-centered applications: use of information technology to promote disease management and wellness. A white paper by the AMIA knowledge in motion working group. J Am Med Inform Assoc 2008;15:8-13.
7. Halamka JD, Mandl KD, Tang PC. Early experiences with personal health records. J Am Med Inform Assoc 2008;15:1-7.
8. Hess R, Bryce CL, Paone S, et al. Exploring challenges and potentials of personal health records in diabetes self-management: implementation and initial assessment. Telemed J E Health 2007;13:509-17.
9. Kaelber DC, Jha AK, Johnston D, Middleton B, Bates DW. A research agenda for personal health records (PHRs). J Am Med Inform Assoc 2008;15:729-36.
10. Wald JS, Middleton B, Bloom A, et al. A patient-controlled journal for an electronic medical record: issues and challenges. Stud Health Technol Inform 2004;107:1166-70.
11. Agarwal R, Anderson C, Zarate J, Ward C. If we offer it, will they accept? Factors affecting patient use intentions of personal health records and secure messaging. J Med Internet Res 2013;15:e43.

12. Holtrop JS, Corser W, Jones G, Brooks G, Holmes-Rovner M, Stommel M. Health behavior goals of cardiac patients after hospitalization. Am J Health Behav 2006;30:387-99.
13. Lau K, Freyer-Adam J, Gaertner B, Rumpf HJ, John U, Hapke U. Motivation to change risky drinking and motivation to seek help for alcohol risk drinking among general hospital inpatients with problem drinking and alcohol-related diseases. Gen Hosp Psychiatry 2010;32:86-93.
14. Sieck CJ, Heirich M, Major C. Alcohol counseling as part of general wellness counseling. Public Health Nurs 2004;21:137-43.
15. Chodosh J, Morton SC, Mojica W, et al. Meta-analysis: chronic disease self-management programs for older adults. Ann Intern Med 2005;143:427-38.
16. Warsi A, Wang PS, LaValley MP, Avorn J, Solomon DH. Self-management education programs in chronic disease: a systematic review and methodological critique of the literature. Arch Intern Med 2004;164:1641-9.
17. Adler-Milstein J, Sarma N, Woskie LR, Jha AK. A comparison of how four countries use health IT to support care for people with chronic conditions. Health Aff 2014;33:1559-66.
18. Archer N, Fevrier-Thomas U, Lokker C, McKibbon KA, Straus SE. Personal health records: a scoping review. J Am Med Inform Assoc 2011;18:515-22.
19. Or CK, Tao D. Does the use of consumer health information technology improve outcomes in the patient self-management of diabetes? A meta-analysis and narrative review of randomized controlled trials. Int J Med Inform 2014;83:320-9.
20. McAlearney AS, Sieck CJ, Hefner JL, et al. High touch and high tech (HT2) proposal: transforming patient engagement throughout the continuum of care by engaging patients with portal technology at the bedside. JMIR Res Protoc 2016;5:e221.
