# Supplementary material for: Differences by Race in Outcomes of an In-Person Training Intervention on Use of an Inpatient Portal: A Secondary Analysis of a Randomized Clinical Trial
Source: JAMA Netw Open. 2024 Apr 4;7(4):e245091. doi: 10.1001/jamanetworkopen.2024.5091 (PMC11192182; doi:10.1001/jamanetworkopen.2024.5091)
Supplement: Supplement 3. — Data Sharing Statement [file jamanetwopen-e245091-s003.pdf]

Data Sharing Statement

Data
Data available: Yes
Data types: Deidentified participant data
How to access data: Data may be requested from the PI via a request to ht2study@osumc.edu
When available: With publication

Supporting Documents
Document types: Statistical/analytic code
How to access documents: Supporting documents may be requested from the PI via a request to ht2study@osumc.edu
When available: With publication

Additional Information
Who can access the data: Open to all requesters who provide a methodologically sound proposal whose use has also been approved by an independent review committee.
Types of analyses: There are no a priori limits. Limits are based on approved proposals.
Mechanisms of data availability: Secure Data transfer. Interested parties will be required to complete an institutional Data Use Agreement.
